# Early versus delayed defunctioning ileostomy closure after low anterior resection for rectal cancer: a meta-analysis and trial sequential analysis of safety and functional outcomes

**DOI:** 10.1007/s00384-022-04106-w

**Published:** 2022-02-21

**Authors:** Mauro Podda, Federico Coccolini, Chiara Gerardi, Greta Castellini, Michael Samuel James Wilson, Massimo Sartelli, Daniela Pacella, Fausto Catena, Roberto Peltrini, Umberto Bracale, Adolfo Pisanu

**Affiliations:** 1 Department of Surgical Science, University of Cagliari, Cagliari, Italy; 2 Emergency Surgery Unit, Cagliari University Hospital “D. Casula”, Azienda Ospedaliero-Universitaria Di Cagliari, Cagliari, Italy; 3 Department of General, Emergency and Trauma Surgery, Pisa University Hospital, Pisa, Italy; 4 Centro di Politiche Regolatorie in Sanità, Istituto di Ricerche Farmacologiche “Mario Negri” - IRCSS -, Milano, Italy; 5 Unit of Clinical Epidemiology, IRCCS Istituto Ortopedico Galeazzi, Milan, Italy; 6 Department of General Surgery, Forth Valley Royal Hospital, Larbert, UK; 7 Department of General and Emergency Surgery, Macerata General Hospital, Macerata, Italy; 8 Department of Public Health, University of Naples Federico II, Naples, Italy; 9 Department of General, Emergency and Trauma Surgery, Bufalini Hospital, Cesena, Italy; 10 Department of Public Health, Minimally Invasive General and Oncological Surgery Unit, University of Naples Federico II, Naples, Italy; 11 Department of Surgical Science, University of Cagliari, Policlinico Universitario “D. Casula”, Azienda Ospedaliero-Universitaria Di Cagliari, SS 554, Km 4,500, 09042 Monserrato, Italy

**Keywords:** Early ileostomy closure, Late ileostomy closure, Rectal cancer, Low anterior resection, Meta-analysis, Trial sequential analysis

## Abstract

**Purpose:**

We performed a systematic review and meta-analysis with trial sequential analysis (TSA) to answer whether early closure of defunctioning ileostomy may be suitable after low anterior resection.

**Methods:**

MEDLINE, EMBASE, and the Cochrane Central Register of Controlled Trials were searched, up to October 2021, for RCTs comparing early closure (*EC* ≤ 30 days*)* and delayed closure (*DC* ≥ 60 days) of defunctioning ileostomy. The risk ratio (RR) with 95% CI was calculated for dichotomous variables and the mean difference (MD) with 95% CI for continuous variables. The GRADE methodology was implemented for assessing Quality of Evidence (QoE). TSA was implemented to address the risk of random error associated with sparse data and/or multiple testing.

**Results:**

Seven RCTs were included for quantitative synthesis. 599 patients were allocated to either *EC* (*n* = 306) or *DC* (*n* = 293). *EC* was associated with a higher rate of wound complications compared to *DC* (RR 2.56; 95% CI 1.33 to 4.93; *P* = 0.005; *I*^2^ = 0%, QoE High), a lower incidence of postoperative small bowel obstruction (RR 0.46; 95% CI 0.24 to 0.89; *P* = 0.02; *I*^2^ = 0%, QoE moderate), and a lower rate of stoma-related complications (RR 0.26; 95% CI 0.16 to 0.42; *P* < 0.00001; *I*^2^ = 0%, QoE moderate). The rate of minor low anterior resection syndrome (LARS) (RR 1.13; 95% CI 0.55 to 2.33; *P* = 0.74; *I*^2^ = 0%, QoE low) and major LARS (RR 0.80; 95% CI 0.59 to 1.09; *P* = 0.16; *I*^2^ = 0%, QoE low) did not differ between the two groups. TSA demonstrated inconclusive evidence with insufficient sample sizes to detect the observed effects.

**Conclusion:**

*EC* may confer some advantages compared with a *DC*. However, TSA advocated a cautious interpretation of the results.

**Prospero Register ID:**

CRD42021276557

**Supplementary Information:**

The online version contains supplementary material available at 10.1007/s00384-022-04106-w.

## Background

Low anterior resection (LAR) with total mesorectal excision is considered the optimal surgical treatment for resectable primary rectal cancer [1, 2]. However, anastomotic leakage (AL) remains the most feared complication after LAR [3], with an incidence of 3 to 25% [4].

In order to protect the colorectal anastomosis and reduce the severity of pelvic sepsis associated with AL, a reversible faecal diversion through a temporary defunctioning ileostomy is usually fashioned during LAR [5–9], especially for patients at increased risk for AL, such as those with locally advanced tumours, located in the low rectum, and treated with neoadjuvant radiotherapy [10–12].

Defunctioning ileostomies are typically reversed after a time interval of 8–12 weeks, during which time 20 to 74% of patients will experience ileostomy-related complications [13–16].

As the incidence of stoma-related complications seems to increase with the time to ileostomy closure [17], it has been suggested that early ileostomy closure could reduce the length of exposure to stoma-related morbidity, improve quality of life, and reduce stoma-related costs, with no detriment to the integrity of the colorectal anastomosis [18–20].

Some recent meta-analyses have suggested that early ileostomy closure is effective and safe in selected patients [21–24]. However, others reported that early closure increased the rate of post-operative complications [25]. Similarly, several randomised controlled trials (RCTs) have reported promising results [18, 26–29], whereas other RCTs had to be prematurely terminated due to safety concerns [30, 31]. Over the last year, a new RCT has been published that has provided further evidence concerning the safety and feasibility of early closure of defunctioning ileostomy, which has not been included in any of the systematic reviews published to date [30].

Given the above, we decided to perform an updated systematic review and meta-analysis with trial sequential analysis (TSA) to answer whether early closure of temporary diverting ileostomy is suitable for selected patients without post-operative complications after LAR.

## Material and methods

### Study objective

We performed a systematic review and meta-analysis with TSA of RCTs with the aim to systematically review the currently available evidence on early defunctioning ileostomy closure (≤ 30 days after index operation, *EC*) in patients who underwent LAR for rectal cancer in terms of safety, long-term functional outcomes and costs, and to compare the above with delayed timing of ileostomy closure (> 60 days after index operation, *DC*) using the meta-analytic method and The Grading of Recommendations Assessment, Development and Evaluation (GRADE) approach [32].

This meta-analysis was conducted according to the recommendations of the 2020 updated Preferred Reporting Items for Systematic reviews and Meta-analyses (PRISMA) guidelines [33] and the *Cochrane Handbook for Systematic Reviews of Interventions* [34].

All stages of study identification, selection, quality assessment, and data extraction were performed independently by two reviewers (MP and AP). Inconsistencies were resolved through discussion between the two reviewers until a consensus was reached. This meta-analysis adheres to AMSTAR II criteria (A MeaSurement Tool to Assess Systematic Reviews) [35].

### Inclusion and exclusion criteria

The clinical question according to the PICOS framework was:

- (P) Population: patients with temporary defunctioning ileostomy after LAR for rectal cancer.
- (I) Intervention: early ileostomy closure (*EC*); ≤ 30 days after ileostomy was performed.
- (C) Comparison: delayed ileostomy closure (*DC*); ≥ 60 days after ileostomy was performed.
- (O) Outcomes, as reported in the included studies: intraoperative and post-operative outcomes of safety and feasibility; functional outcomes; quality of life.
- (S) Studies: RCTs comparing the two approaches.

The following studies were excluded: emergency ileostomy in case of AL, ileostomy performed during other types of surgery other than LAR for cancer, studies that involved paediatric patients (< 18 years of age), studies that included other types of enteral stomas such as colostomy, proximal/mid small bowel stomas, or where the type of stoma was not specified, systematic or narrative reviews, meta-analyses, abstracts, case reports, study protocols, non-human studies, non-comparative studies. In addition, studies in which closure time was influenced by factors (i.e. chemotherapy, the occurrence of complications after LAR, delays due to operating theatre unavailability) other than patient’s randomisation and allocation into a predefined study group were excluded.

### Study identification

MEDLINE (via PubMed), EMBASE, and the Cochrane Central Register of Controlled Trials were systematically searched for any relevant human clinical study comparing *EC* and *DC* of defunctioning ileostomy after LAR for rectal cancer. Grey literature searches were conducted on ClinicalTrials.gov, Google Scholar, CORE, Grey Literature Report, and Open Grey. Reference lists of relevant studies were searched manually, and the “related articles” function in PubMed was used. In addition, the reference list from the selected articles was also scrutinised. The search strategy combined medical subject headings (MeSH) and keywords, using the terms of “Ileostomy”, “Defunctioning stoma”, “Early”, “Closure”, “Reversal”, “Proctectomy”, “Low Anterior Resection”, “Rectal Cancer”, and “Colorectal Cancer”. The detailed search strategy is accessible in the registered protocol (PROSPERO: CRD42021276557). No restrictions were placed on publication status or language. Full-text articles in languages other than English with a title/abstract indicating fulfilment of the eligibility criteria were translated electronically. Literature was searched from inception to October 31, 2021.

### Study selection

The studies identified by the search strategy were subsequently selected based on title, abstract, and full-text review by two independent reviewers (MP and AP) in Rayyan web app for systematic reviews (https://www.rayyan.ai/).

RCTs comparing *EC (Intervention*) and *DC (Control*) as the most appropriate timing for ileostomy reversal after LAR for rectal cancer were included in the meta-analysis. Whenever there was an overlap in patient cohorts of two or more studies, and no difference in the study period was reported, the most recent report was included in the pooled analysis. Secondary analyses from the included RCTs were also included if they focused on outcomes other than those analysed in the primary study.

### Data extraction

A double-blinded procedure was performed to increase the accuracy of the data extracted, which resulted in high and satisfactory inter-observer agreement (*Kappa* = 0.92).

The following individual data were independently extracted using standardised extraction forms (Excel 2019, Microsoft Corporation®). Demographic and baseline characteristics collected for each report included the following predefined data: (1) study identifier (first author, nationality, year of publication, clinical trials centres, study period, inclusion criteria, exclusion criteria, index operation, intervention, comparator, analysed outcomes, follow-up times); (2) baseline characteristics of the enrolled patients (sex, Age, BMI, comorbidities, type of surgery and technique of primary anastomosis construction, indication for colorectal resection, neoadjuvant therapy); (3) clinical outcomes (post-operative morbidity, mortality, leak of rectal or ileal anastomosis, unplanned reoperations, operative time, post-operative length of hospital stay, time to start chemotherapy); (4) morbidity outcomes analysis (Clavien-Dindo complications ≥ 3, post-operative ileus/small bowel obstruction, wound complications, post-operative intra-abdominal abscess, post-operative enterocutaneous fistula, bleeding, stoma-related complications, anastomotic stenosis, other medical complications, hospital readmission); (5) quality of life, functional outcomes, and costs (Gastrointestinal Quality of Life Index (GQLI), EORTC QLQ-C30 Quality of Life, low anterior resection syndrome, Memorial Sloan Kettering Cancer Centre Bowel Function Instrument).

### Outcomes measures

The following primary outcomes were analysed:

- Overall post-operative morbidity: defined as any complication occurring during the hospital stay, within 30 days, or 12 months after ileostomy closure.
  The following secondary outcomes were analysed:
- *Morbidity outcomes stratified per different type of complication*: leak of rectal anastomosis, leak of ileal anastomosis, Clavien-Dindo ≥ 3 complications, post-operative ileus/small bowel obstruction, wound complications, post-operative intra-abdominal abscess, post-operative enterocutaneous fistula, bleeding, stoma-related complications, anastomotic stenosis, other medical complications, hospital readmission, unplanned reoperations;
- *Functional outcomes*: Low anterior resection syndrome (LARS), Memorial Sloan Kettering Cancer Centre Bowel Function Instrument (MSKCC-BFI);
- *Quality of life*: Gastrointestinal Quality of Life Index (GQLI), EORTC QLQ-C30 Quality of Life;
- *Costs*: Stoma-related costs, total costs;
- *Operative time*;
- *Post-operative length of hospital stay*;
- *Time to start chemotherapy*.

Anastomotic leak was defined as any leak from the rectal anastomosis detected clinically and/or radiologically, including intra-abdominal abscess, enterocutaneous fistula, and anastomotic insufficiency [36]. Stoma-related complications were defined as the presence of any complications attributable to the presence of the ileostomy (i.e. dermatitis, parastomal infection, dehydration from high stoma output, renal insufficiency, stenosis, retraction, necrosis, prolapse, skin irritation, parastomal hernia) occurring between the index operation and stoma closure [37].

### Statistical analysis

Variables for meta-analysis were considered if they were reported by at least two RCTs. All statistical analyses were performed using Reviewer Manager software (Reviewer Manager – RevMan – version 5.4.1, Sept. 2020, The Nordic Cochrane Centre, Cochrane Collaboration, www.training.cochrane.org) and RevMan Web 2020 [38]. The relative risk (RR) with 95% confidence interval (95% CI) was calculated for dichotomous variables and the mean difference (MD) with 95% CI for continuous variables. The point estimate of the RR value was considered statistically significant if the 95% CI did not cross the value at null hypothesis (RR = 1). The point estimate of the MD value was considered statistically significant if the 95% CI did not cross the value at null hypothesis (MD = 0). Statistical heterogeneity of the results across studies was assessed using the Higgins inconsistency index *I*^2^ and Chi-square test. A Chi-square test *P* < 0.10 and an *I*^2^ value of 50 to 90% were considered indicative of substantial heterogeneity. In addition to statistical heterogeneity, both clinical (variability in the baseline characteristics of the patients, interventions and outcomes studied) and methodological (variability in the study methods and risk of bias) heterogeneity were considered to inform the decision to use the fixed- or random-effects model. Fixed-effects model (Mantel–Haenszel) was used if substantial heterogeneity was absent, whereas a random-effects model was implemented for meta-analysis if substantial heterogeneity was found, according to the method of DerSimonian and Laird [39]. The analyses were performed using the Trial Sequential Analysis software version 0.9.5.10 Beta (The Copenhagen Trial Unit, Centre for Clinical Intervention Research, The Capital Region, Copenhagen University Hospital – Rigshospitalet, 2021).

### Trial sequential analysis

The TSA was performed to address the risk of random error associated with sparse data and/or multiple testing which can affect cumulative meta-analysis analyses, and to assess whether further trials need to be conducted [40]. Indeed, the TSA can inform regarding how much more information is required to get a conclusive answer about the effect of the intervention versus its comparator and this is represented by the distance between the accrued information and the required information. We applied the TSA on the following patient-centred dichotomous outcomes, which are relevant for clinical practice due to their consequences for patient management, and are the most frequently reported in the literature: post-operative morbidity as the primary outcome, leak of rectal anastomosis, and unplanned reoperations as secondary outcomes.

We estimated the diversity-adjusted required information size (DARIS) based on the proportion of patients with an outcome in the control group, calculating the average control group event proportion, an alpha (type I error) of 5%, a beta (type II error) of 20%, and diversity model-based. Both the naïve and TSA-adjusted confidence intervals were reported. We set the conservative trial monitoring boundaries by Lan-DeMets-O’Brien-Fleming as the α-spending function [41, 42]. We calculated the cumulative *Z*-curve (the series of *Z*-statistics after each consecutive trial) of each cumulative meta-analysis and plotted it against the above monitoring boundaries according to the random-effects models. The crossing of the cumulative *Z*-curve into the trial sequential monitoring boundary for benefit indicates that a sufficient level of evidence has been reached, and no further trials may be needed to demonstrate the superiority of the intervention. Otherwise, when the cumulative *Z*-curve does not cross any of the trial sequential monitoring boundaries, there is likely insufficient evidence to reach a conclusion, and additional trials may be required [43].

We developed two scenarios according to two different risk ratio reduction (RRR) values. *Scenario 1* was based on RRR values pragmatically chosen to represent a conservative intervention effect: 10% for post-operative morbidity outcome, 1% leak of rectal anastomosis, 5% unplanned reoperations [21–25, 44]. *Scenario 2* represented a sensitivity analysis where an RRR of 25% was chosen to represent an optimistic intervention effect for all outcomes. The results are presented as the TSA figures with corresponding legends and interpretations.

### Planned sensitivity and subgroup analyses

Sensitivity analyses of post-operative morbidity and other clinically relevant outcomes were performed, depending on:

- The different primary bowel diseases (rectal cancer versus rectal cancer plus others);
- Depending on some concerns of bias according to the ROB-2 evaluation.

Furthermore, given that substantial differences in clinical settings, especially regarding the timing of stoma closure within the *EC* group, the effect of the timing of *EC* for all outcomes was analysed by a subgroup analysis of *very early closure* (defined as closure ≤ 14 days), *early closure* (defined as closure between 15 and 30 days), in comparison with *DC* (defined as closure ≥ 60 days).

### Risk of bias and quality of evidence assessment

The risk of bias of the included RCTs was independently assessed by two authors (MP and AP) using the Cochrane Risk of Bias Tool version 2 (RoB 2) [45]. In addition, the Grading of Recommendations Assessment, Development and Evaluation (GRADE) methodology was implemented for assessing the quality of evidence (QoE) [46, 47], which was reported in the results with Summary of Findings Tables.

## Results

### Study selection

**Fig. 1:**
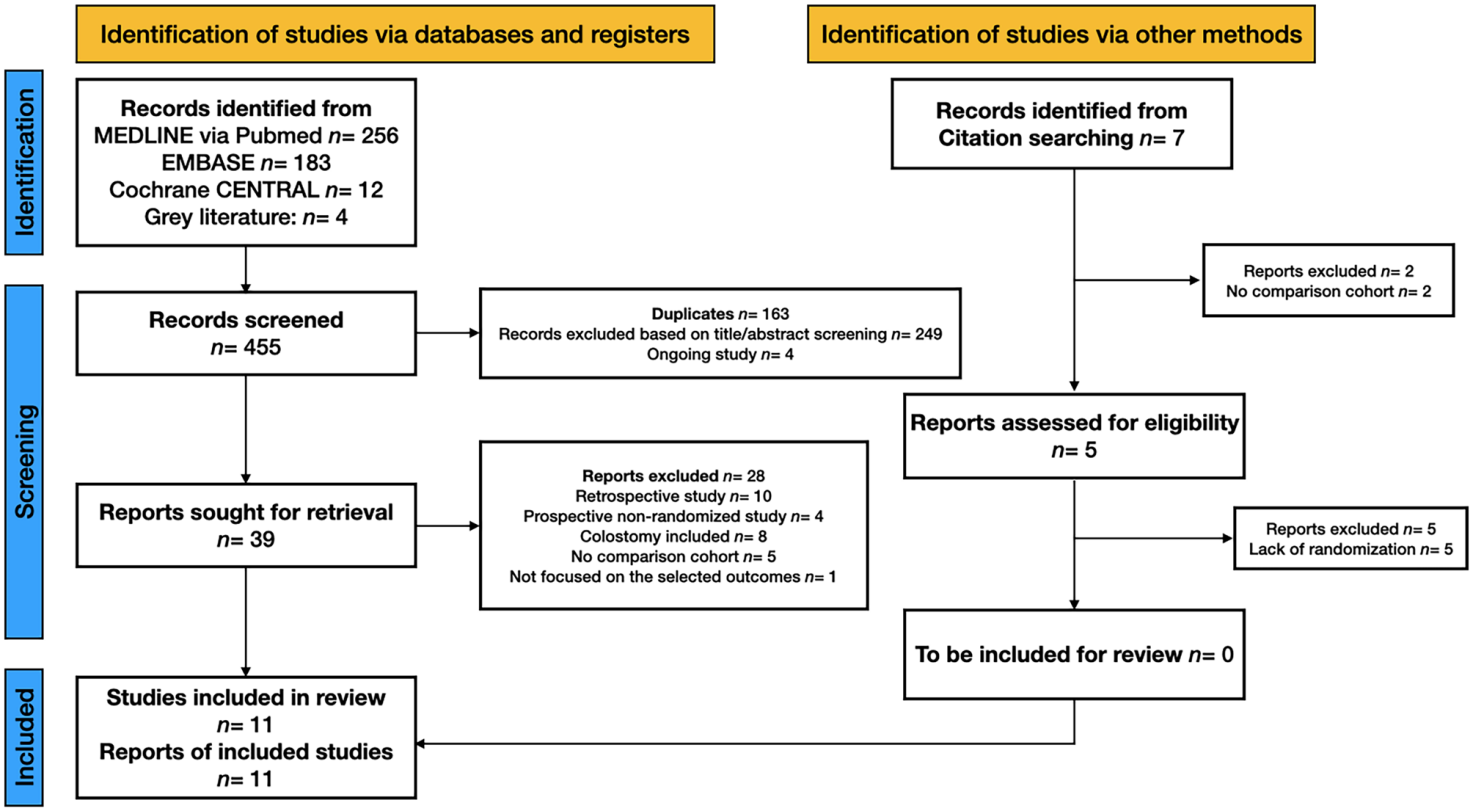
Search results and selection of included studies. PRISMA 2020 flow diagram

A total of 455 records were identified through database searching. Seven more references were identified by searching lists of retrieved studies. After removing 163 duplicates and four study protocols of ongoing studies, 288 records had their titles and abstracts evaluated. This resulted in 39 articles suitable for full-text review, where ten were retrospective studies, four were prospective non-randomised studies, eight included colostomy closure procedures, five had no comparative cohort, and one did not focus on the selected outcomes of interest. Finally, 11 RCTs were included for quantitative synthesis, of which seven were primary studies [18, 26–31] and four were secondary analyses of the primary RCTs [48–51] (Table 1). In total, 599 patients were allocated to either *Early ileostomy closure* (*EC*) (*n* = 306) or *Delayed ileostomy closure* (*DC*) (*n* = 293). General patient characteristics of the patients as reported in the studies are shown in Supplementary Table 1. Supplementary Table 2 shows the evidence report of each included study. Figure 1 reports the flow diagram of the study selection phases and reported excluded reasons for full texts not being eligible.

**Table 1:** Main characteristics of the included studies

Reference	Nationality	Centers	Overall risk of bias (ROB 2)	Study period and design	Inclusion criteria	Exclusion criteria	Index operation
Alves et al. [18] (RCT)	France	Clichy Marseille Paris Boulogne	Low risk	2001–2004	All patients over 18 years old with benign or malignant disease requiring elective rectal resection with a low anastomosis (7 cm or less above the anal verge)	Contraindications to early closure of the temporary loop ileostomy, such as signs of active infection or organ failure in the postoperative period, or radiological signs of anastomotic leakage evident at a water-soluble contrast examination through the temporary loop ileostomy performed 7 days after surgery	Rectal resection with low colorectal, coloanal or ileoanal anastomosis
Lasithiotakis et al. [26] (RCT)	UK	York	Some concerns	2004–2007	All consecutive patients (under a single colorectal consultant) having a defunctioning ileostomy during a low rectal or anal anastomosis and with satisfactory gastrografin enema on postoperative day 6	Patients on steroids, at high cardiorespiratory risk, and those experiencing any postoperative complication (primary operation) or a radiological leak	Rectal resection with low rectal or anal anastomosis
Danielsen et al. [27] (RCT) Park et al. [48]^1^ Park et al. [49]^2^ Keane et al. [50]^**1**^	Denmark Sweden	Herlev-Copenaghen Gothenburg	Low risk	2011–2015	> 18 y/o patients without any clinical sign of postoperative complications after the index operation (infections or clinical signs of leakage) were invited to participate and after informed consent went through further investigation with a contrast CT scan or a flexible endoscopy or both. This was performed 6 to 8 days after stoma creation to make sure that no patients with signs of anastomotic leakage were included	Patient with a leak of contrast outside the rectum at the CT scan with a water-soluble contrast medium. Patients with diabetes, patients being treated with steroids, patients with linguistic difficulties, and patients with expected low compliance	Rectal resection with TME (total mesorectal excision) for cancer
Klek et al. [29] (RCT)	Poland	Skawina Krakow	Some concerns	2016–2017	Patients ≥ 18 years of age who had undergone anterior resection of the rectum with protective loop ileostomy for rectal adenocarcinoma	Colorectal malignancy other than rectal adenocarcinoma, protective loop ileostomy performed for other indications (part of treatment for postoperative complication), lack of informed consent	Low anterior resection of the rectum
Gallyamov et al. [28] (RCT)	Russia	NR	Some concerns	NR	Total or partial mesorectal excision for rectal cancer with formation of a defunctioning ileostomy	Radiological or endoscopic signs of anastomotic insufficiency, diabetes, steroid treatment, expected low compliance	Total or partial mesorectal excision
Bausys et al. [31] (RCT) Dulskas et al. [51]^3^	Lithuania	Vilnius	Low risk	2011–2017	Patients over 18 years old with rectal cancer were screened and included in the study after the elective rectal resection with a temporary loop ileostomy. Patients were included in the study on the 10^th^ postoperative day if they did not meet any of the exclusion criteria	Contraindications to the closure of temporary ileostomy, such as radiological/endoscopic or clinical signs of colorectal anastomosis insufficiency, also general contraindications for surgery such as signs of active infection or organ failure, which would contraindicate ileostomy closure 30 days after creation	Elective rectal resection with colorectal anastomosis (lower than 6 cm from the anal verge) with a temporary loop ileostomy
Elsner et al. [30] (RCT)	Switzerland	NR	Low risk	2007–2014	Patients undergoing low anterior resection (LAR) for rectal cancer were eligible for participation. Inclusion criteria were age > 18 years, planned anastomosis at 5 cm or less from the anal verge with consecutive fecal diversion via loop ileostomy, and obtained informed consent	Pregnancy, allergy to contrast agent, limited contractual capability, and abdominopelvic or severe non-surgical complications	Open low anterior resection with anastomosis at 5 cm or less from the anal verge for rectal cancer

Reference	Nationality	Centers	Intervention	Comparator	Outcomes	Delay until stoma closure (days)	Follow-up times
Alves et al. [18] (RCT)	France	Clichy Marseille Paris Boulogne	Early closure (EC): stoma closure on day 8	Delayed closure (DC): stoma closure on day 60	*Primary:* morbidity and mortality within 90 days of proctectomy *Secondary:* total hospital stay, functional results, quality of life at 12 months	EC: 8 (8–10) DC: 66 (62–69) *Median (range)*	1, 2, 3, 6, and 12 months after the rectal resection; functional results were assessed at 3 and 12 months after the first operation. Quality of life (Gastrointestinal Quality of Life Index) was measured before and at 12 months after operation
Lasithiotakis et al. [26] (RCT)	UK	York	Early closure (EC): stoma closure on day 7	Delayed closure (DC): stoma closure after an interval of 8 weeks	Duration of the operation, ease of reversal of stoma and closure of the abdominal wall, postoperative complications, costs associated with stoma care	EC: 8 (2) DC: 57 (38) *Median (IQR)*	NR
Danielsen et al. [27] (RCT) Park et al. [48]^1^ Park et al. [49]^2^ Keane et al. [50]^**1**^	Denmark Sweden	Herlev-Copenaghen Gothenburg	Early closure (EC): stoma closure on days 8–13	Delayed closure (DC): stoma closure after > 12 weeks	*Primary:* mean number of complications after index operation and up to 12 months *Secondary:* the proportion of patients with at least one complication with Clavien-Dindo IIIa, IIIb, IVa, IVb, or V after index surgery and up to 12 months, and the mean number of stoma-related complications after index surgery and up to 12 months	EC: 11 (8–152) DC: 148 (64–665) *Median (range)*	At the time of stoma closure, and 3, 6, and 12 months after index surgery
Klek et al. [29] (RCT)	Poland	Skawina Krakow	Early closure (EC): stoma closure on the 14^th^ day after the discharge after the primary resection	Delayed closure (DC): stoma closure after 30 days after the termination of the adjuvant chemotherapy (usually approximately 7 months after the primary resection	*Primary:* Complication rate *Secondary:* Impact of early closure on the start of postoperative adjuvant anticancer treatment	EC: 17.3 ± 1.5 DC: 278.6 ± 89.1 *Mean* ± *SD*	NR
Gallyamov et al. [28] (RCT)	Russia	NR	Early closure (EC): ileostomy closure on days 8–13 after rectal excision	Delayed closure (DC): ileostomy closure > 12 weeks after rectal excision	Postoperative morbidity, duration of reconstructive surgery	EC: 11 (8–21) DC: 148 (64–265) *Median (range)*	NR
Bausys et al. [31] (RCT) Dulskas et al. [51]^3^	Lithuania	Vilnius	Early closure (EC): ileostomy closure 30 days after creation	Delayed closure (DC): ileostomy closure 90 days after creation	*Primary:* Number of postoperative complications after the ileostomy closure and 30 days afterward *Secondary:* Hospitalization time and 30 days readmission rate	EC: 34 (29–47) DC: 92 (80–157) *Median (range)*	30^th^ postoperative day after ileostomy closure
Elsner et al. [30] (RCT)	Switzerland	NR	Early closure (EC): ileostomy closure 2 weeks after creation	Delayed closure (DC): ileostomy closure 12 weeks after creation	*Primary:* Quality of life (QoL), assessed by the GQLI questionnaire, 6 weeks after LAR (about 4 weeks after stoma closure in the early closure group and about 6 weeks before stoma closure in the late group, respectively) *Secondary:* QoL 6 weeks after LAR, but assessed by the EORTC-QLQ-C30 questionnaire, and QoL 4 months after LAR, assessed by GQLI and EORTC-QLQ-C30 questionnaire; intraoperative feasibility (operation time, blood loss, tendency of oozing, parastomal and intraabdominal adhesions, difference in diameters between the two stoma limbs), postoperative recovery (need of parenteral fluids, first defecation, full oral intake, length of hospital stay), morbidity/safety and the general feasibility based on the check of the colonic anastomosis	EC: 15 (10–104) DC: 89 (76–128) *Median (range)*	6 weeks and 4 months after low anterior resection

### Study characteristics

Considerable variability was found among the included studies concerning the definition and the time frame of *EC* and *DC*. Some studies defined *EC* as the closure of the stoma within 8 days after LAR [18, 26], whereas in others, patients allocated to *EC* had their ileostomy closed within 2 weeks of LAR [27, 28]. Delay until stoma closure in the *EC* group was 17 days in the study by Klek et al. [29] and 15 days in the study by Elsner et al. [30]. Finally, in the study by Bausys et al. patients in the *EC* group had undergone stoma closure 30 days after creation [31]. Significant heterogeneity also existed in terms of delay until ileostomy closure in the *DC* group, with timing for closure that varied between 57 [26] and 278 days [29] after stoma creation. Most studies included patients with rectal cancer as the sole indication for LAR. Two studies [18, 26] included benign, borderline, and inflammatory bowel diseases. Different follow-up times were reported, ranging from 30 days [31] to 12 months [18, 27]. Two RCTs were prematurely terminated for safety reasons [30, 31].

### Primary outcome: Overall post-operative morbidity

**Fig. 2:**
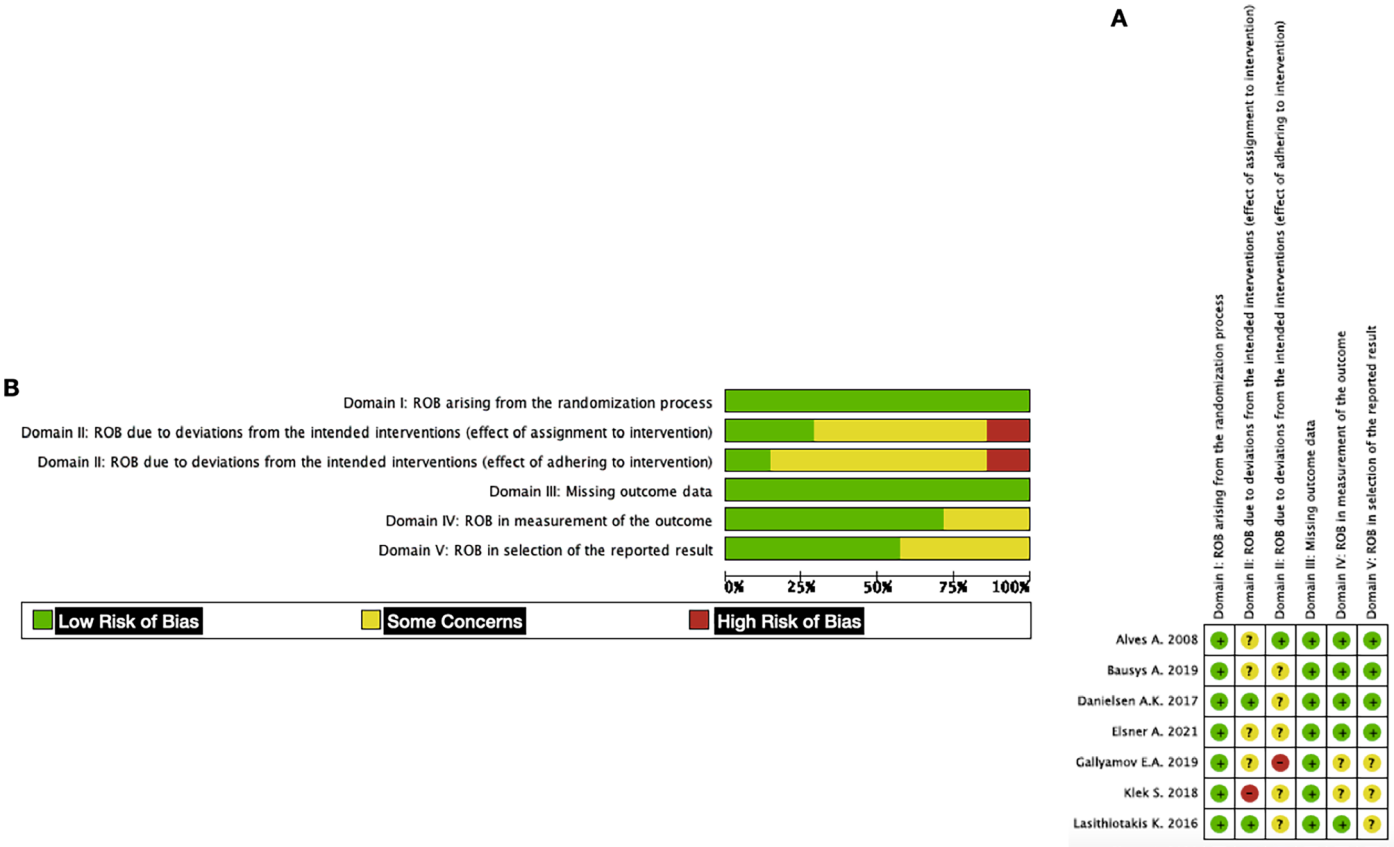
**A** Risk of bias summary. **B** Risk of bias graph

No statistically significant difference was found between the two groups in terms of overall postoperative morbidity (7 studies, 599 patients; RR 0.99, 95% CI 0.78 to 1.78; *P* = 0.95; *I*^2^ = 37%, fixed-effects; QoE low; test for subgroup differences: Chi^2^ = 5.59, *P* = 0.02, *I*^2^ = 82.1%) (Fig. 2) (Supplementary Table 3).

### Secondary outcomes: Morbidity outcomes stratified per different types of complication

**Fig. 3:**
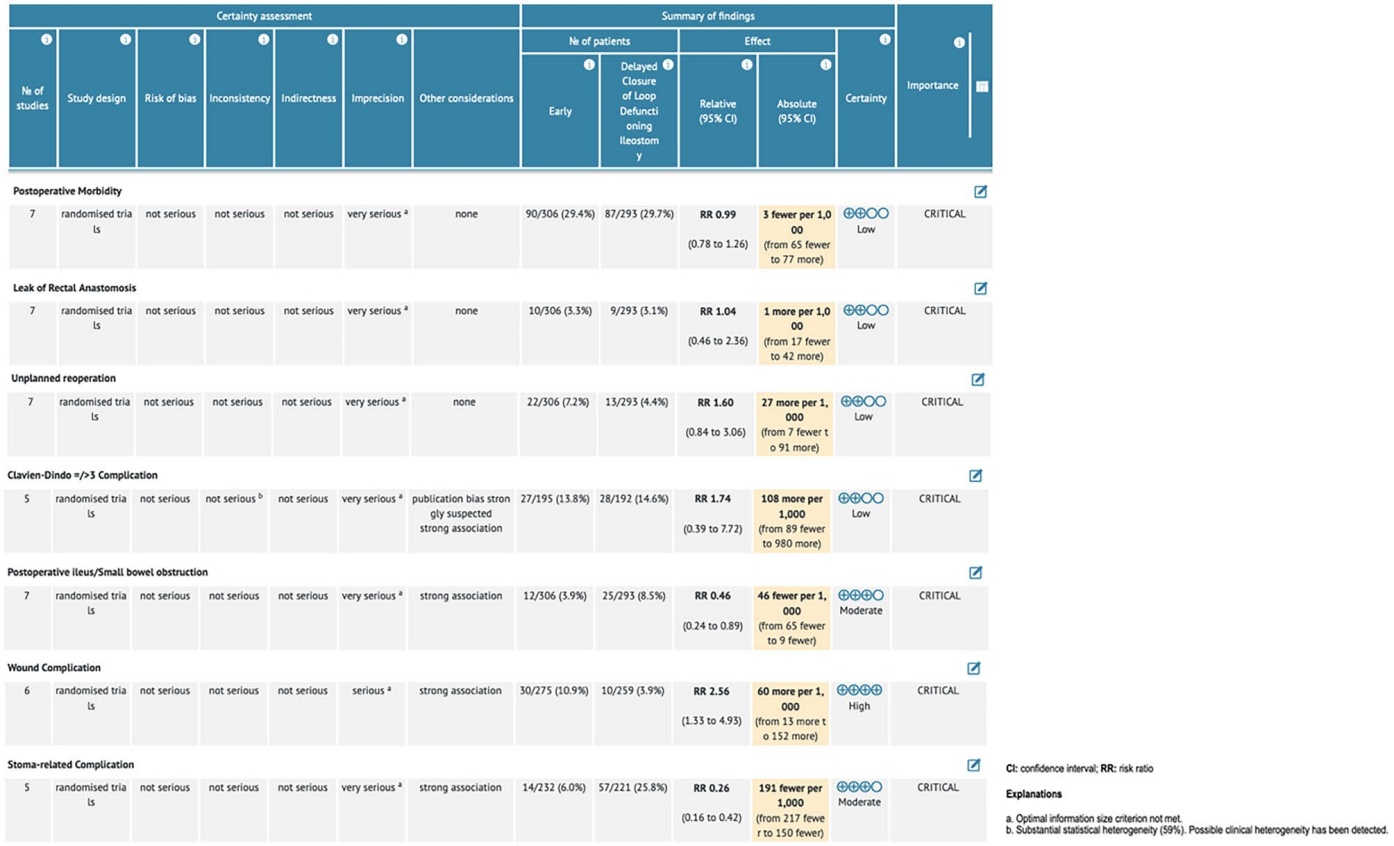
Early compared to delayed defunctioning ileostomy closure after low anterior resection for rectal cancer. GRADE evidence profile

*EC* and *DC* showed equivalent results in terms of leak of the rectal anastomosis (7 studies, 599 patients; RR 1.04; 95% CI 0.46 to 2.36; *P* = 0.92; *I*^2^ = 0%, Fixed-effects; QoE low; test for subgroup differences: Chi^2^ = 2.63, *P* = 0.11, *I*^2^ = 61.9%) (Fig. 3), leak of the ileal anastomosis (6 studies, 413 patients; RR 4.52; 95% CI 0.54 to 37.78; *P* = 0.16; *I*^2^ = 0%, fixed-effects; QoE low; test for subgroup differences: not applicable), unplanned reoperation (7 studies, 599 patients; RR 1.60; 95% CI 0.84 to 3.06; *P* = 0.15; *I*^2^ = 0%, fixed-effects; QoE low; test for subgroup differences: Chi^2^ = 2.19, *P* = 0.14, *I*^2^ = 54.4%), Clavien-Dindo ≥ 3 complications (5 studies, 387 patients; RR 1.74; 95% CI 0.39 to 7.72; *P* = 0.46; *I*^2^ = 59%, random-effects; QoE low; test for subgroup differences: Chi^2^ = 5.81, *P* = 0.02, *I*^2^ = 82.8%) (Fig. 3), postoperative intra-abdominal abscess (6 studies, 543 patients; RR 1.32; 95% CI 0.44 to 3.92; *P* = 0.62; *I*^2^ = 0%, fixed-effects; QoE low; test for subgroup differences: Chi^2^ = 1.37, *P* = 0.24, *I*^2^ = 27.2%), postoperative enterocutaneous fistula (3 studies, 338 patients; RR 4.06; 95% CI 0.70 to 23.51; *P* = 0.12; *I*^2^ = 0%, fixed-effects; QoE low; test for subgroup differences: Chi^2^ = 0.09, *P* = 0.76, *I*^2^ = 0%), postoperative bleeding (6 studies, 534 patients; RR 0.59; 95% CI 0.08 to 4.38; *P* = 0.60; *I*^2^ = 0%, fixed-effects; QoE low; test for subgroup differences: Chi^2^ = 0.24, *P* = 0.62, *I*^2^ = 0%), anastomotic stenosis (6 studies, 534 patients; RR 1.49; 95% CI 0.25 to 9.01; *P* = 0.66; *I*^2^ = 28%, fixed-effects; QoE low; test for subgroup differences: not applicable), other medical complications (6 studies, 534 patients; RR 1.42; 95% CI 0.69 to 2.93; *P* = 0.34; *I*^2^ = 0%, fixed-effects; QoE low; test for subgroup differences: Chi^2^ = 0.30, *P* = 0.59, *I*^2^ = 0%), and hospital readmission (2 studies, 152 patients; RR 1.36; 95% CI 0.40 to 4.63; *P* = 0.62; *I*^2^ = 0%, fixed-effects; QoE low; test for subgroup differences: not applicable).

**Fig. 4:**
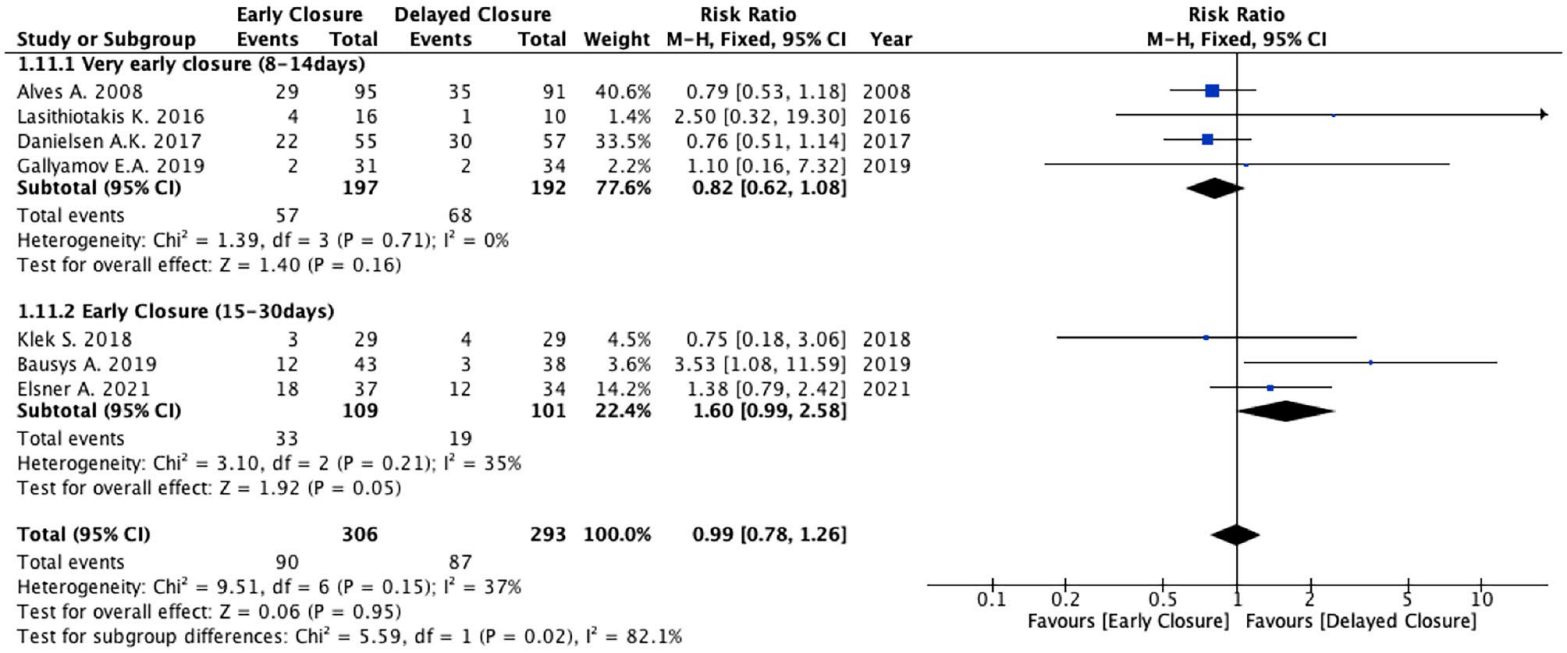
Meta-analysis of overall post-operative morbidity

**Fig. 5:**
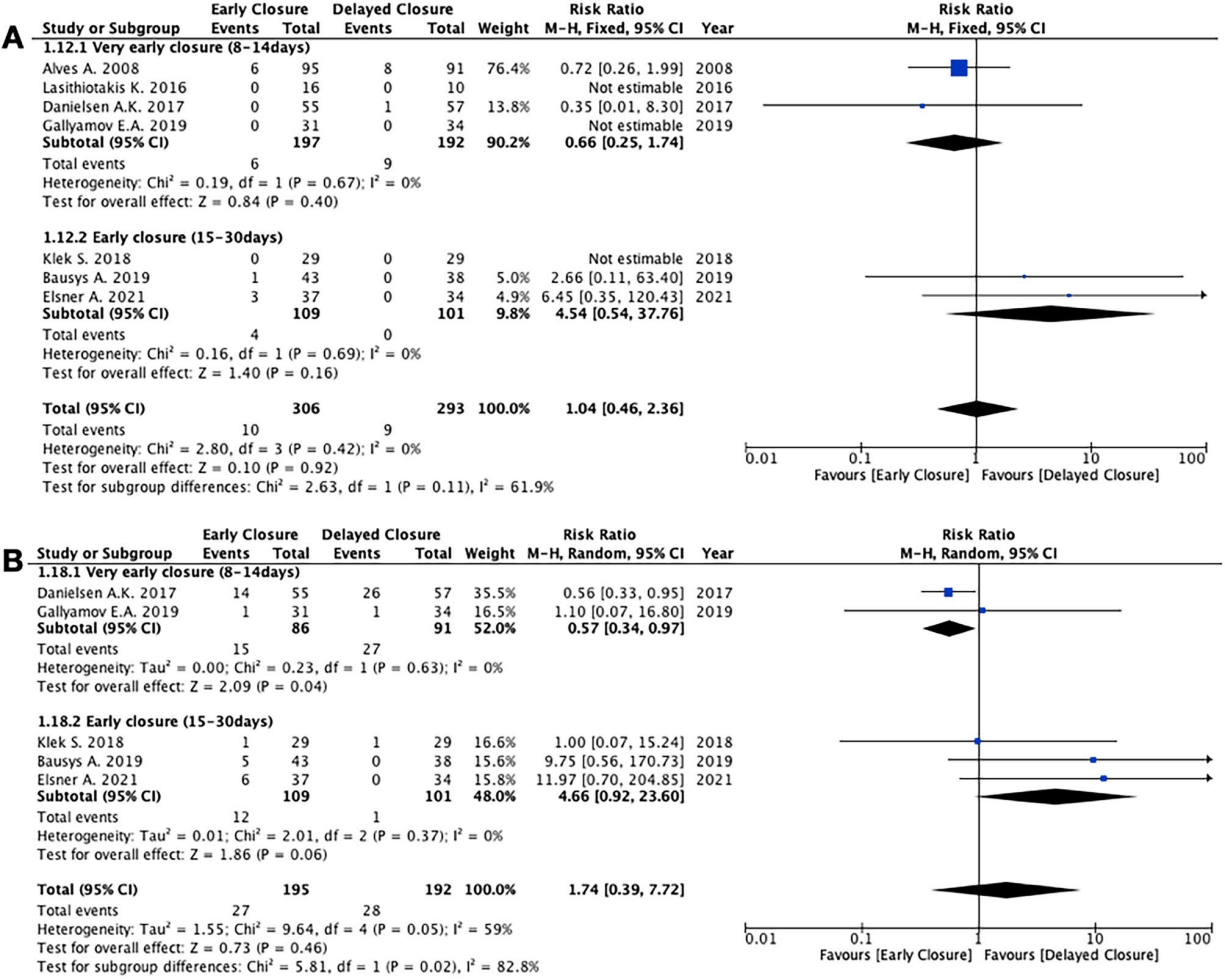
Meta-analyses of specific morbidity outcomes. **A** Leak of rectal anastomosis. **B** Clavien-Dindo complications ≥ 3

*EC* was associated with a higher rate of wound complications compared to *DC* (6 studies, 534 patients; RR 2.56; 95% CI 1.33 to 4.93; *P* = 0.005; *I*^2^ = 0%, fixed-effects; QoE high; test for subgroup differences: Chi^2^ = 1.67, *P* = 0.20, *I*^2^ = 40.2%) (Fig. 4), a lower incidence of postoperative small bowel obstruction (7 studies, 599 patients; RR 0.46; 95% CI 0.24 to 0.89; *P* = 0.02; *I*^2^ = 0%, fixed-effects; QoE moderate; test for subgroup differences: Chi^2^ = 3.08, *P* = 0.08, *I*^2^ = 67.6%) (Fig. 5), and a lower rate of stoma-related complications (5 studies, 453 patients; RR 0.26; 95% CI 0.16 to 0.42; *P* < 0.00001; *I*^2^ = 0%, fixed-effects; QoE moderate; test for subgroup differences: Chi^2^ = 0.05, *P* = 0.82, *I*^2^ = 0%) (Fig. 4) (Supplementary Table 4).

### Secondary outcomes: Quality of life and functional outcomes

**Fig. 6:**
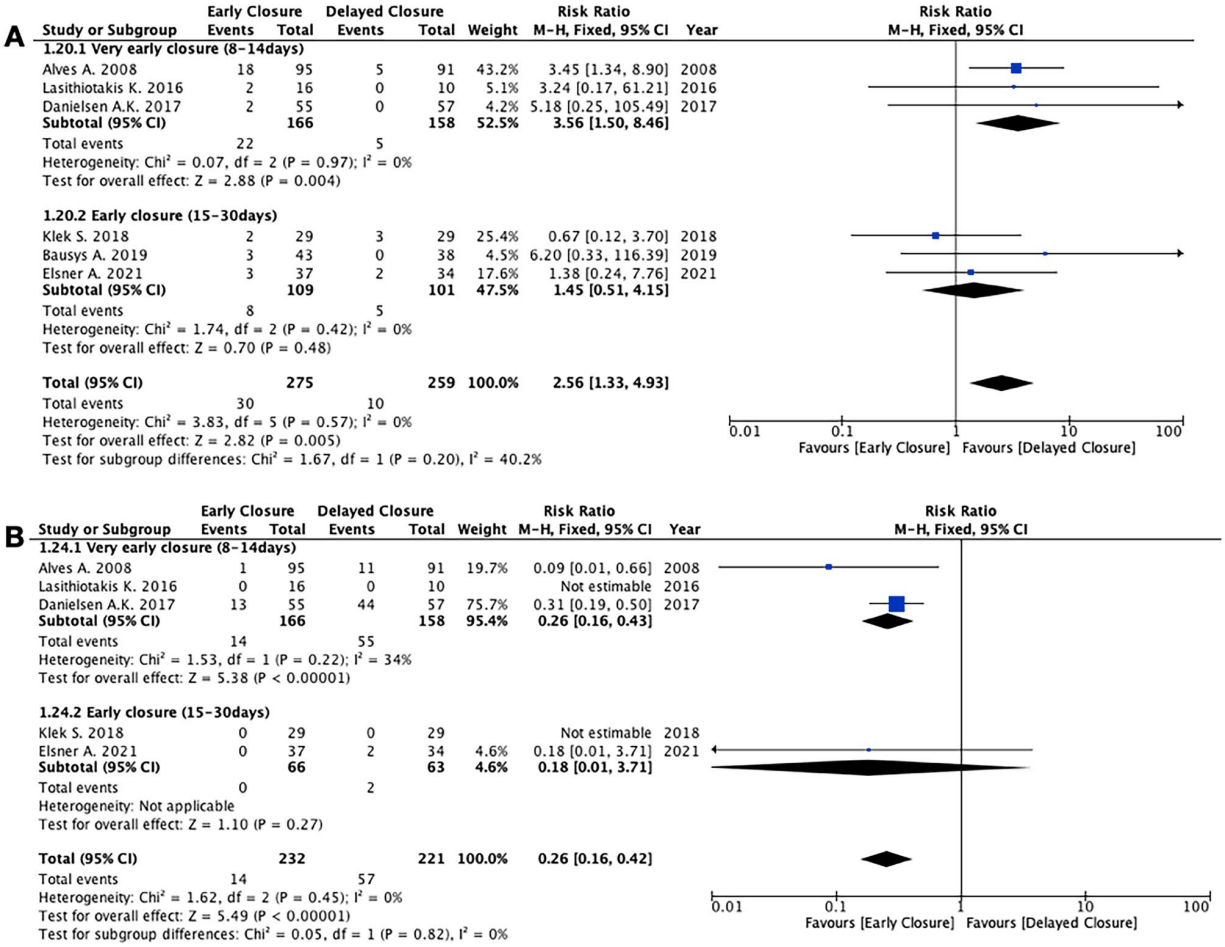
Meta-analyses of specific morbidity outcomes. **A** Wound complications. **B** Stoma-related complications

*EC* and *DC* showed equivalent functional outcomes in terms of quality of life, calculated with the GQLI (2 studies, 257 patients; MD 1.22; 95% CI − 2.80 to 5.24; *P* = 0.55; *I*^2^ = 44%, fixed-effects; QoE low; test for subgroup differences: not applicable) and with the EORTC QLQ-C30 Quality of Life (2 studies, 148 patients; MD 0.23; 95% CI − 3.01 to 3.48; *P* = 0.89; *I*^2^ = 0%, fixed-effects; QoE low; test for subgroup differences: not applicable). The pooled analyses showed that the rates of minor LARS (2 studies, 133 patients; RR 1.13; 95% CI 0.55 to 2.33; *P* = 0.74; *I*^2^ = 0%, fixed-effects; QoE low; test for subgroup differences: not applicable) and major LARS (2 studies, 133 patients; RR 0.80; 95% CI 0.59 to 1.09; *P* = 0.16; *I*^2^ = 0%, fixed-effects; QoE low; test for subgroup differences: not applicable) did not differ between the two groups (Fig. 6) (Supplementary Table 5).

### Secondary outcomes: Operative time and post-operative length of hospital stay

Operative time in the *EC* group ranged between 20 (median, IQR 13) [26] and 130 min (median, range 60 − 240) [30], compared with 40 (median, IQR 9) [26] to 110 min (median, range 60 − 257) [30] in the *DC* group. Overall length of hospital stay in the *EC* group ranged between 14 (median, range 11 − 42) [27] and 28 days (median, range 17 − 77) [30], compared with 14 (median, range 7 − 44) [27] to 27 days (median, range 17 − 87) [30] in the *DC* group. Length of hospital stay after ileostomy closure showed wide variability, ranging between 4 (median, range 2 − 27) [27] and 7 days (median, range 6 − 9) [31] in the *EC* group and 4 (median, range 2 − 21) [28] to 6 days (median, range 6 − 7) [31] in the *DC* group (Supplementary Table 3). Quantitative synthesis was not performed for either operative time or length of hospital stay due to the lack of data reported as mean and standard deviation. The Hozo method to convert median and range into mean and standard deviation [52] was not used as the data distribution in the primary RCTs might be skewed, so the approach mentioned above may not be appropriate [34].

### Secondary outcomes: Costs

Costs were evaluated by Lasithiotakis et al. [26], Klek et al. [29], and Park et al. [49]. However, quantitative synthesis was not performed due to the high methodological heterogeneity regarding outcome metrics and settings.

The study by Lasithiotakis et al. showed that *EC* was superior compared to *DC* in terms of costs of stoma care (27 £ median, 9 IQR versus 311 £ median, 108 IQR). In the study by Park et al. the difference in mean cost per patient was $4060 in favour of *EC*. Taking protocol-driven examinations into account, the sensitivity analysis resulted in an overall difference in mean cost per patient of $3608 in favour of *EC*. Similarly, in the study by Klek et al., a health care cost reduction in favour of *EC* was demonstrated: 152.9 ± 16.3 versus 2413.1 ± 759 (cost of stoma bags/treatment period) (Supplementary Table 5).

### Secondary outcomes: Time to start chemotherapy

Time to start chemoradiotherapy was analysed by Klek et al. [29]. Mean time to start adjuvant treatment was 38.7 ± 5.7 and 33.2 ± 5.8 days in the *EC* and *DC* groups, respectively (*P* < 0.001) (Supplementary Table 3).

### Subgroup analyses

Two subgroup meta-analyses (very early closure, defined as closure 8 − 14 days, and early closure, defined as closure between 15 and 30 days) showed that the rate of post-operative morbidity was slightly higher in the early closure group compared to the *DC* group (RR 1.60; 95% CI 0.99 to 2.58; *P* = 0.05; *I*^2^ = 35%, fixed-effects), although without statistical significance. Similarly, the unplanned reoperation rate showed an increased rate in the early closure group compared to the *DC* (RR 4.19; 95% CI 0.91 to 19.30; *P* = 0.07; *I*^2^ = 0% fixed-effects). The rate of Clavien-Dindo ≥ 3 complications was lower in the very early closure group compared to the *DC* group (RR 0.57; 95% CI 0.34 to 0.97; *P* = 0.04; *I*^2^ = 0%, fixed-effects), whereas the rate of wound complication was comparable between the early closure group and the *DC* group (RR 1.45; 95% CI 0.51 to 4.15; *P* = 0.48; *I*^2^ = 0%, Fixed-effects).

### Sensitivity analyses

Sensitivity analyses excluding the RCTs by Lasithiotakis et al. [26] and Alves et al. [18] that also included patients with benign disease in the study population found an equivalent rate of post-operative small bowel obstruction comparing the very early closure (8 − 14 days) and the *DC* groups (RR 0.64; 95% CI 0.09 to 4.76; *P* = 0.66; *I*^2^ = 0%, fixed-effects). Sensitivity analyses excluding studies with some concerns of bias according to the ROB-2 evaluation found similar outcomes between the *EC* and *DC* groups in terms of overall post-operative morbidity, although in the subgroup comparison between the early closure group (15 − 30 days) and the *DC* group a statistically significant difference was found in favour of *DC* (RR 1.82; 95% CI 1.08 to 3.04; *P* = 0.02; *I*^2^ = 53%, random-effects). Equivalent outcomes were also found regarding the leak of rectal anastomosis and unplanned reoperation. Wound complication rate was higher in the *EC* compared to the *DC* group (4 studies, 450 patients; RR 3.20; 95% CI 1.50 to 6.86; *P* = 0.003; *I*^2^ = 0%, fixed-effects; test for subgroup differences: Chi^2^ = 0.24, *P* = 0.62, *I*^2^ = 0%), with the majority of wound complications occurring in the very early closure group (8 − 14 days) (RR 3.60; 95% CI 1.46 to 8.89; *P* = 0.005; *I*^2^ = 0%, fixed-effects). The rate of small bowel obstruction remained lower in the *EC* compared to the *DC* group (4 studies, 450 patients; RR 0.41; 95% CI 0.20 to 0.84; *P* = 0.01; *I*^2^ = 13%, fixed-effects; test for subgroup differences: Chi^2^ = 2.37, *P* = 0.12, *I*^2^ = 57.9%), with a greater effect in favour of the very early closure group (8 − 14 days) (RR 0.24; 95% CI 0.08 to 0.70; *P* = 0.009; *I*^2^ = 18%, fixed-effects).

### Trial sequential analysis

For post-operative morbidity, scenario 1, using an RRR of 10% and a control event rate of 29.7%, we obtained a DARIS (Diversity Adjusted Required Information Size) of 16,330 patients. The cumulative *Z*-curve did not surpass either the traditional monitoring boundary or the trial sequential monitoring boundaries. TSA graph was not available due to insufficient information use (3.67%). Using an RRR of 25%, scenario 2, we obtained a DARIS of 2478 patients: the cumulative *Z*-curve did not surpass either the traditional monitoring boundary or the trial sequential monitoring boundaries with an alpha-spending adjusted CI of 0.61–1.63.

For leak of rectal anastomosis, scenario 1, using an RRR of 1% and a control event rate of 3%, we obtained a DARIS of 10,102,024 patients. The cumulative *Z*-curve did not surpass either the traditional or the trial sequential monitoring boundaries. Using an RRR of 25% and a control event rate of 3%, scenario 2, we obtained a DARIS of 14,267 patients: the cumulative *Z*-curve did not surpass either the traditional or the trial sequential monitoring boundaries. TSA graphs were unavailable for both scenarios due to insufficient information use (< 4%).

**Fig. 7:**
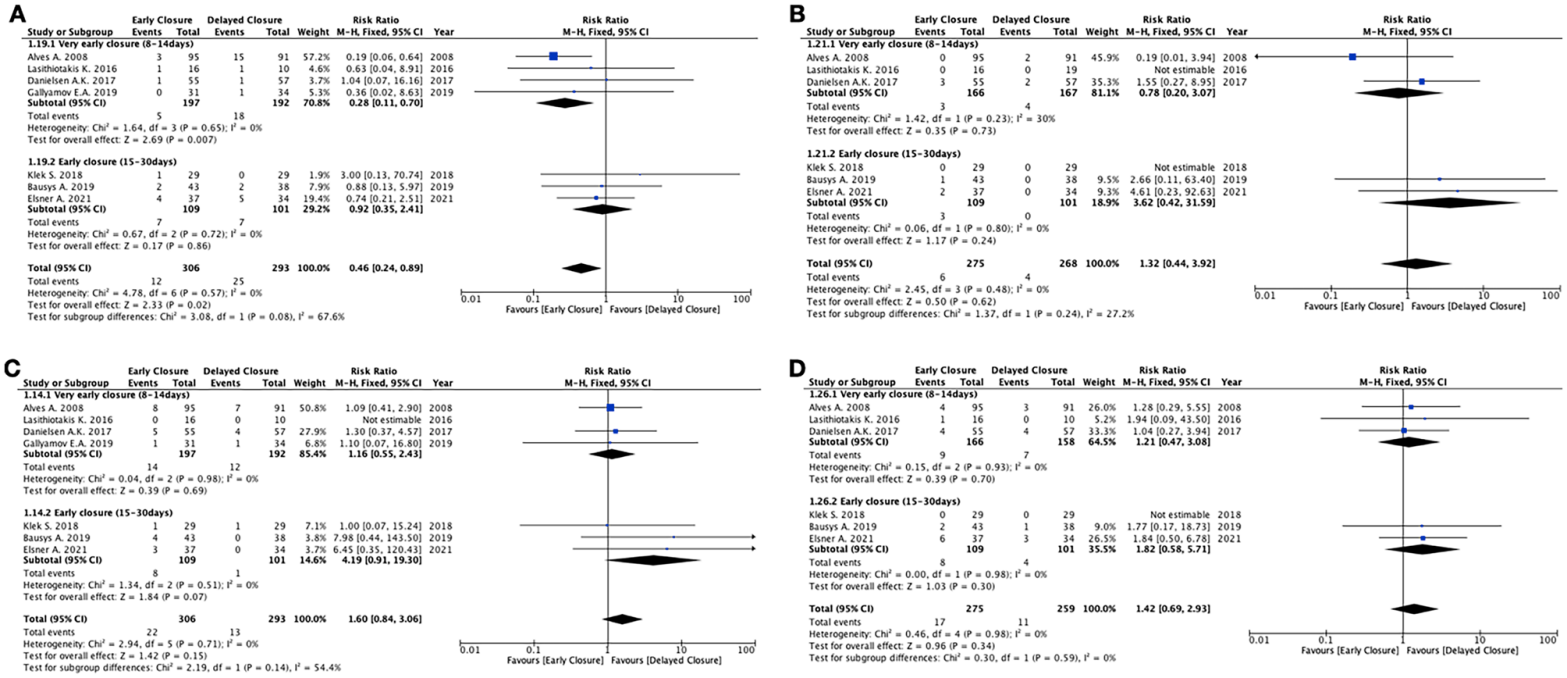
Meta-analyses of specific morbidity outcomes. **A** Post-operative small bowel obstruction. **B** Post-operative intra-abdominal abscess. **C** Unplanned reoperations. **D** Other medical complications

For unplanned reoperations, scenario 1, using an RRR of 5% and a control event rate of 4%, we obtained a DARIS of 294,169 patients. The cumulative *Z*-curve did not surpass either the traditional or the trial sequential monitoring boundaries. TSA graph was not available due to little information use (0.19%). Using an RRR of 25% and a control event rate of 4%, scenario 2, we obtained a DARIS of 10,604 patients: the cumulative *Z*-curve did not surpass either the traditional or the trial sequential monitoring boundaries with an alpha-spending adjusted CI of 0.11–22.54 (Fig. 7) (Supplementary Table 6).

### Risk of bias and certainty of the evidence assessment

**Fig. 8:**
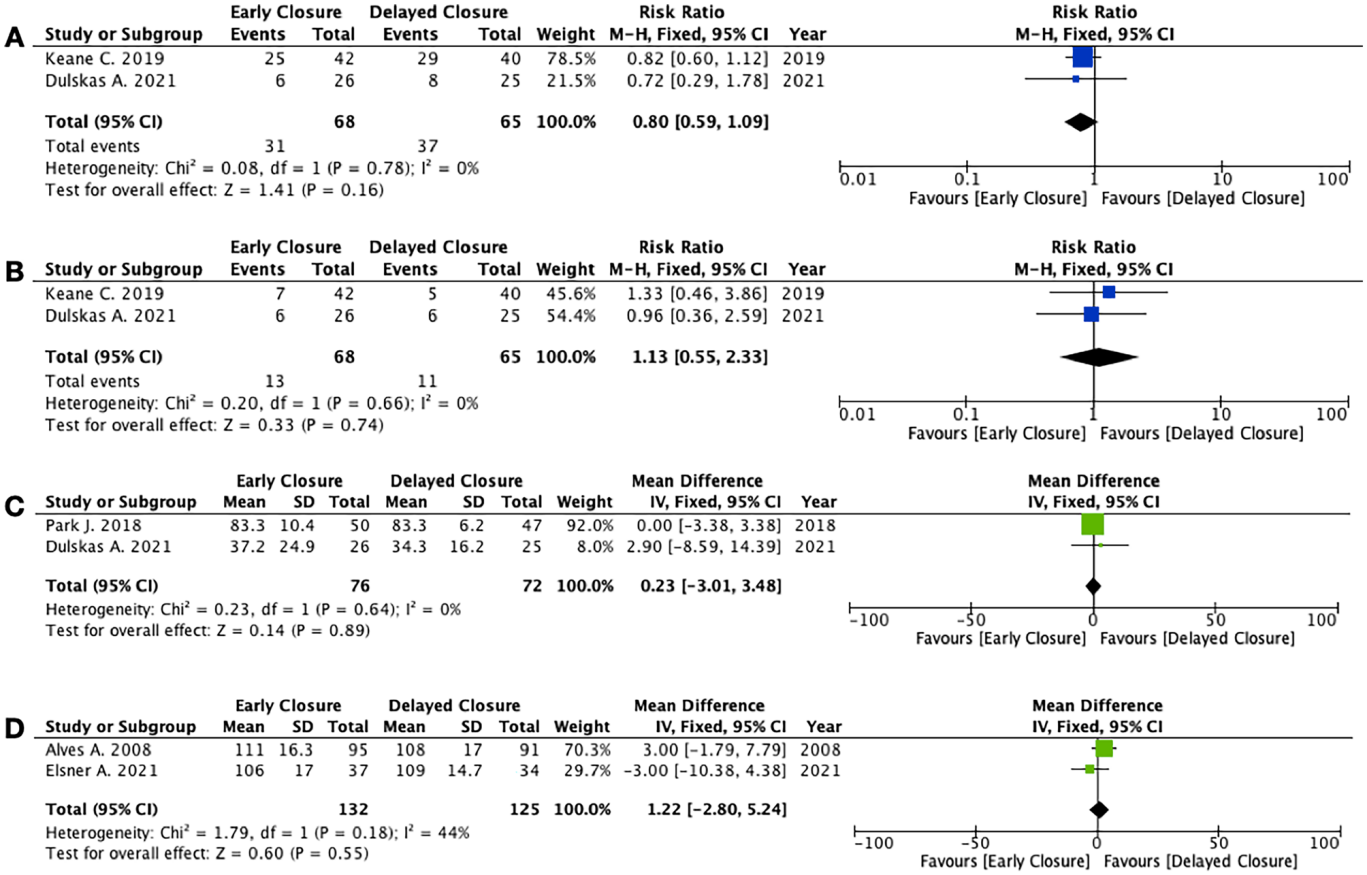
Meta-analyses of functional outcomes and quality of life. **A** Major LARS. **B** Minor LARS. **C** Quality of life EORTC (EORTC QLQ-C30 Quality of Life). **D** Quality of life GQLI (Gastrointestinal Quality of Life Index)

**Fig. 9:**
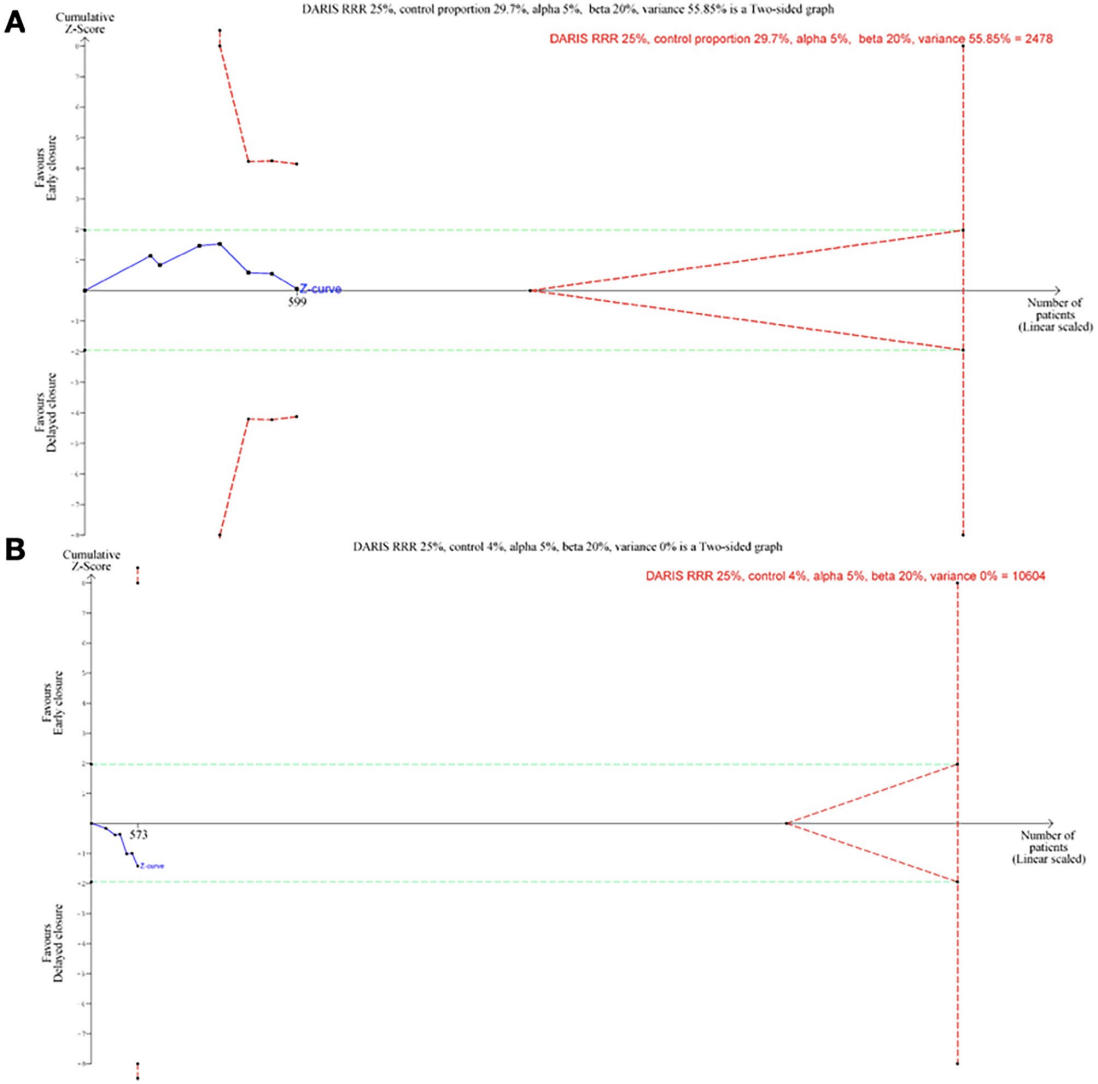
Trial sequential analysis of **A** post-operative morbidity (scenario 2): trial sequential analysis of *EC* vs *DC* for post-operative morbidity. The diversity adjusted information size (DARIS) was calculated based on a control event proportion of 29.7%, a relative risk reduction (RRR) of 25%, an alpha (**a**) of 0.05, a beta (**b**) of 0.20, and diversity **D** of 55.85. **B** Unplanned reoperation (scenario 2): trial sequential analysis of EC vs DC for unplanned reoperation. The diversity adjusted information size (DARIS) was calculated based on a control event proportion of 4%, a relative risk reduction (RRR) of 25%, an alpha (**a**) of 0.05, a beta (**b**) of 0.20, and diversity **D** of 0

The quality of the seven included primary RCTs was evaluated according to the Cochrane Risk of Bias Tool (RoB) version 2 (Fig. 8). Four RCTs were considered at low risk of bias [18, 27, 30, 31], whereas three RCTs contained some concerns [26, 28, 29] (Supplementary Table 1). According to the GRADE criteria, the overall QoE was high only for wound complications. It was moderate for post-operative ileus/small bowel obstruction and stoma-related complication but low for post-operative morbidity, leak of rectal anastomosis, unplanned reoperation, and Clavien-Dindo complication ≥ 3 (Fig. 9). Potential publication bias was present for Clavien-Dindo complication ≥ 3. Funnel plots have been provided as supplemental digital content (Supp. Digit. Content Fig. 1).

## Discussion

In keeping with previous studies, the current meta-analysis with TSA of RCTs, including data of 599 patients, indicates that patients who had undergone early ileostomy closure within 30 days from LAR for rectal cancer experienced lower occurrence of small bowel obstruction and stoma-related complication but, at the same time, incur a higher rate of ileostomy closure-related wound complications [21–25]. Wound complications after ileostomy closure can be decreased by the implementation of evidence-based recommendations on closure techniques. As reported in the Italian guidelines for the surgical management of enteral stomas in adults, purse-string closure in stoma reversal should be the preferred skin closure technique because it is associated with lower surgical site infection rates compared to other techniques [53]. All patients undergoing enterostomy closure should receive antibiotic prophylaxis. Although various regimens have been described, oral preoperative antibiotics appear to be associated with less morbidity than parenteral antibiotics, similar to findings already reported for colon surgery [54].

Based on the primary pooled analysis, the incidence of post-operative morbidity was similar for patients who underwent early and delayed ileostomy closure ≥ 60 days from LAR. However, the sensitivity analysis that excluded the study by Alves et al. suggested that the higher rate of wound complications in the early closure group was mainly attributable to a closure strategy within 8 days of the primary resection [18].

The subgroup meta-analyses on very early closure (closure 8–14 days) and early closure (closure between 15 and 30 days) showed that the rate of post-operative morbidity was lower if stoma closure was performed between the 8^th^ and the 14^th^ post-operative day after the primary LAR. In contrast, patients who underwent stoma closure between the 15^th^ and the 30^th^ day were exposed to 60% increased risk of post-operative complications compared to the delayed closure. Furthermore, patients in the early ileostomy closure group had 4.2 times increased risk of a second, unplanned reoperation and a 4.6 times increased risk of a Clavien-Dindo ≥ 3 complication compared to the delayed closure group.

In synthesis, our findings suggest that the ideal timing for ileostomy closure should be approximately between days 8 and 14 after construction, provided that a satisfactory CT scan-assessed gastrografin enema or a flexible endoscopy, or both, demonstrates no signs of anastomotic insufficiency. This is based on two primary considerations: the first is that the tensile strength of an anastomosis has been shown to rapidly increase at day 5 and to exceed its initial strength at day 7, and by this time, the vast majority of anastomotic leaks will have occurred [19, 55]. The second is that severe dense adhesions around the ileostomy site tend to form after 2 weeks and up to 6 weeks post-operation [56].

LARS is pragmatically defined as disordered bowel function leading to a detriment in quality of life after rectal resection, which encompasses the vital aspects of the patient experience [57]. The risk of developing major LARS seems higher with a defunctioning ileostomy, and a prolonged time to ileostomy closure seems to reinforce the negative effect on bowel function, suggesting that early reversal should be an essential part of the patient pathway [58]. Similarly, a sub-analysis of the EASY trial showed that patients undergoing early ileostomy closure had fewer problems with soiling and fewer had a permanent stoma [50]. Regarding the analysis of functional outcomes, our meta-analysis did not show any advantages for the early ileostomy closure group in terms of quality of life and incidence of LARS. However, our findings may be hampered by small sample sizes and a high risk of imprecision.

Two years of COVID-19 pandemic have dramatically modified the usual clinical practice. Some scientific surgical societies have recommended a prudent approach towards colorectal anastomosis [59, 60]. The majority of surgical procedures have been performed for emergent or oncological reasons to the detriment of the remaining elective procedures for benign conditions. The pandemic may have caused a delay in enterostomy closure, especially in cases of patients who are candidates for adjuvant chemotherapy, with a prolonged deterioration in quality of life, an increase in number of enterostomies that will never be closed, and increased associated health care costs for the management of this new patient cohort. In this regard, a same-admission ileostomy closure strategy in selected patients [18, 26] can contribute on the one hand to reducing the burden on hospitals caused by new hospitalizations due to the presence of ileostomy-related complications, and on the other hand to guarantee a better quality of life for the patient. Also, ghost ileostomy may be a safe and cost-effective method in patients who underwent LAR with low or medium risk factors for AL [61].

A recent meta-analysis with TSA provided “firm evidence the early closure of ileostomy reduced the incidence of small bowel obstruction and post-operative ileus and required less total operative time, but increased the incidence of surgical site infections compared with late closure of ileostomy” [22]. Though in keeping with the results of the meta-analysis by Cheng et al., our TSA was not able to reach any firm conclusion on either of the two scenarios according to different RRR: the one that represented a conservative and more realistic intervention effect and the one that represented a sensitivity analysis where an RRR of 25% was chosen to represent an optimistic intervention effect for all outcomes.

The current literature has substantial limitations, especially concerning multiple studies with small sample sizes, as highlighted in our TSA. In contrast to other systematic reviews and meta-analyses that provided more optimistic conclusions about the feasibility, efficacy, and safety of early closure of defunctioning ileostomies in selected patients without taking into account the high risk of imprecision and all the risks of drawing firm evidence based on small sample sizes [23], we are more conservative with our conclusions. Even though our results do affirm the view that early ileostomy closure is a valid option, they should be interpreted with caution. In fact, according to the GRADE criteria, the overall quality of evidence was moderate to low for outcomes classified as “critical” in the preplanned analysis.

Further limitations of the present meta-analysis relate to relatively high inter-study heterogeneity for some of the analysed outcomes that required exploration of potential sources. The timing of intervention differed substantially in the study by Bausys et al. [31], where the median time to early ileostomy closure after LAR was 34 days and more than 3 weeks later than the studies by Alves et al. and Lasithiotakis et al. [18, 26]. In the study by Bausys et al. which was prematurely terminated due to a high rate of post-operative morbidity in the early closure group, the authors argue that the high complication rate could be due to a greater technical difficulty of ileostomy closure at day 30, when adhesions have formed, and the inflammatory phase of cicatrisation is still active.

In order to address heterogeneity, we performed several subgroup analyses for very early ileostomy closure and two different sensitivity analyses. Sensitivity analyses excluding studies with some bias concerns according to the ROB-2 evaluation found similar outcomes between the two groups in terms of post-operative morbidity, leak of rectal anastomosis, and unplanned reoperation. Finally, although the concept of late anastomotic leak is still a matter of debate [5, 62], delayed leak of the colorectal anastomosis may occur after the eighth post-operative day despite a negative contrast enema examination or endoscopy. For this reason, we have investigated this specific outcome by excluding the studies by Alves et al. [18] and Lasithiotakis et al. [26] in which the ileostomy was closed between the 7^th^ and the 8^th^ post-operative days in the early closure group, with the result that no difference was found between the two groups in terms of colorectal anastomotic leak.

Several large randomised trials have assessed the outcomes of laparoscopic colectomy compared to conventional open surgery [63–66]. The widespread implementation of laparoscopic surgery in colorectal cancer has been associated with improved postoperative clinical outcomes, including lower blood loss and less postoperative pain, as well as fewer abdominal wall complications, without negatively affecting the oncological outcomes. Conversely, robust evidence on minimally invasive LAR remains lacking [67–69]. Appropriately in light of this, another potential limitation of our study is the lack of information on the type of surgical approach used for the rectal resection. This might have an impact on the results because, after minimal access surgery, it can be expected that adhesions around the ileostomy are less even in the early period after surgery.

This meta-analysis also has significant strengths. It only included RCTs, increasing the likelihood that estimated effects favouring early ileostomy closure for selected patients with an uneventful recovery from the rectal resection may be investigated safely in the future.

Besides the high risk of imprecision, our results should be interpreted with caution, as the patients randomized to early ileostomy closure were selected based on a set of strict criteria where the patients have a low prevalence of coexisting morbidity and have an uneventful post-operative course after rectal resection. Unfortunately, despite the completeness of the analysis, numerous doubts remain, which will be clarified by future studies with a high level of evidence. Should it be decided to transfer our results into daily clinical practice, maximum attention should be paid to carefully select patients, both regarding the general clinical status and the effective healing of the colorectal anastomosis.

## Conclusions

Findings of this meta-analysis with TSA indicate that a narrow window of chance for early closure might exist between the 8^th^ and the 14^th^ post-operative days following LAR.

Preliminary results suggest that very early closure in selected patients is feasible in the absence of radiological or endoscopic signs of anastomotic insufficiency, and may confer some advantages compared with delayed closure after 2 months. The results of our TSA should be interpreted with caution, especially in terms of leak of the colorectal anastomosis, post-operative morbidity, and unplanned reoperations. Therefore, future research should be conducted in the context of randomized controlled trials to determine the effectiveness of early ileostomy closure definitively.

## Supplementary Information

Below is the link to the electronic supplementary material.

Supplementary file1 (Suppl. Digit. Content. Fig.1. Funnel plots. **A** Morbidity **B** Leak of rectal anastomosis **C** Unplanned reoperation **D** Operative time **E** Clavien-Dindo III-IV complication **F** Post-operative small bowel obstruction **G** Wound complication **H** Post-operative intra-abdominal abscess **I** Stoma-related complication TIFF 144 KB)

Supplementary file2 (Suppl. Digit. Content. Table 1. Baseline characteristics of the patients DOC 36 KB)

Supplementary file3 (Suppl. Digit. Content. Table 2. Study characteristics - Evidence assessment DOC 332 KB)

Supplementary file4 (Suppl. Digit. Content. Table 3. Clinical Outcomes of patients following early and delayed ileostomy closure DOC 36 KB)

Supplementary file5 (Suppl. Digit. Content. Table 4. Morbidity Outcomes of patients following early and delayed ileostomy closure DOC 34 KB)

Supplementary file6 (Suppl. Digit. Content. Table 5. Quality of Life, Functional Outcomes and Costs DOC 27 KB)

Supplementary file7 (Digit. Content. Table 6. Results of the Trial Sequential Analysis DOC 22 KB)

## Data Availability

Template data collection forms, data extracted from included studies, data used for all meta-analyses and trial sequential analysis, and any other materials used in the present research are available on request from the corresponding author (MP).
