# Supplementary figures and images for: Early versus delayed defunctioning ileostomy closure after low anterior resection for rectal cancer: a meta-analysis and trial sequential analysis of safety and functional outcomes
Source: Int J Colorectal Dis. 2022 Feb 21;37(4):737–56. doi: 10.1007/s00384-022-04106-w (PMC8860143; doi:10.1007/s00384-022-04106-w)

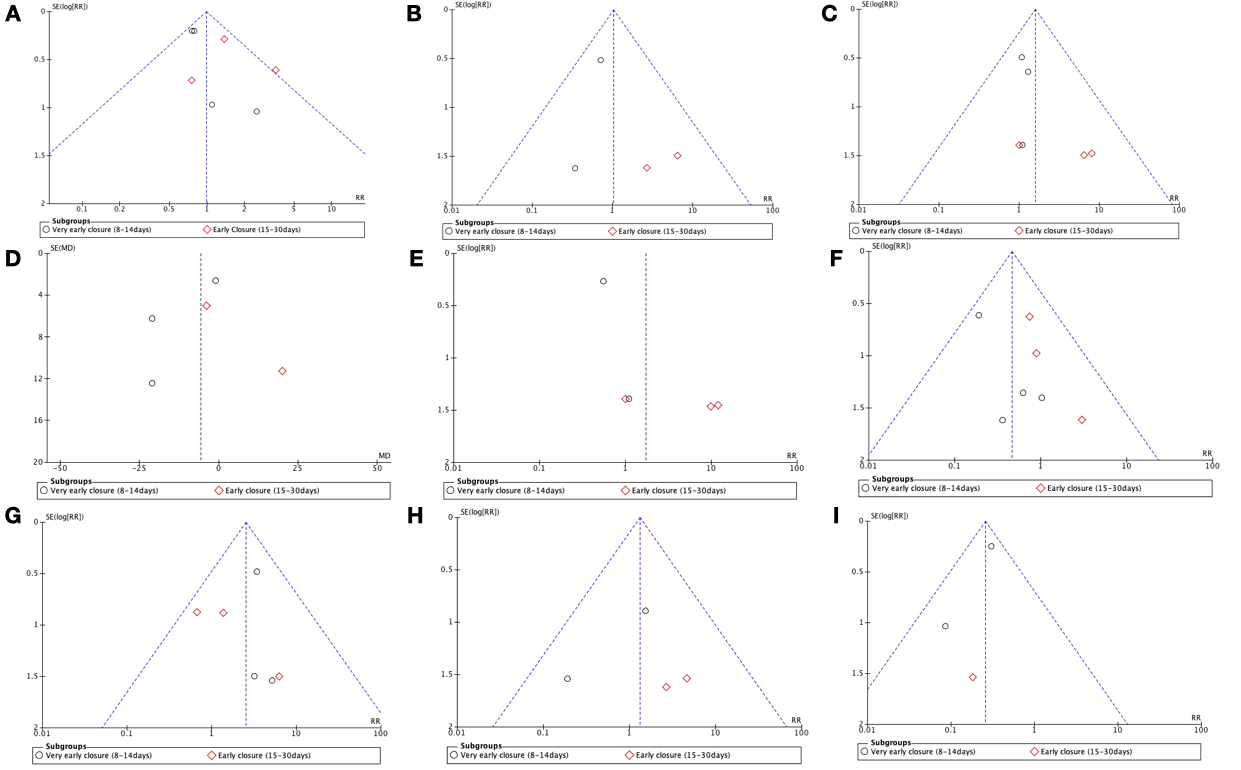

Supplement: Supplementary file 1 — Supplementary file1 (Suppl. Digit. Content. Fig.1. Funnel plots. A Morbidity B Leak of rectal anastomosis C Unplanned reoperation D Operative time E Clavien-Dindo III-IV complication F Post-operative small bowel obstruction G Wound complication H Post-operative intra-abdominal abscess I Stoma-related complication TIFF 144 KB) [file 384_2022_4106_MOESM1_ESM.tiff]
