# Supplementary material for: Early versus delayed defunctioning ileostomy closure after low anterior resection for rectal cancer: a meta-analysis and trial sequential analysis of safety and functional outcomes
Source: Int J Colorectal Dis. 2022 Feb 21;37(4):737–56. doi: 10.1007/s00384-022-04106-w (PMC8860143; doi:10.1007/s00384-022-04106-w)
Supplement: Supplementary file 2 — Supplementary file2 (Suppl. Digit. Content. Table 1. Baseline characteristics of the patients DOC 36 KB) [file 384_2022_4106_MOESM2_ESM.doc]

**Supplementary Table 1. Baseline characteristics of the patients**

| **Reference** | **Patients** | | **Sex (M:F)** | | **Age (years) Median (range) Median (IQR) Mean ± SD** | | **BMI (Kg/m^2^) Mean ± SD** | | **Comorbidities N. (%)** | | **Rectal Anastomosis N. (%)** | | **Primary Bowel Disease** | | **Neoadjuvant Therapy N. (%)** | |
| --- | --- | --- | --- | --- | --- | --- | --- | --- | --- | --- | --- | --- | --- | --- | --- | --- |
|  | **Early** | **Delayed** | **Early** | **Delayed** | **Early** | **Delayed** | **Early** | **Delayed** | **Early** | **Delayed** | **Early** | **Delayed** | **Early** | **Delayed** | **Early** | **Delayed** |
| **Alves A. 2008** | 95 | 91 | 44:51 | 42:49 | 58±11.8 | 56±10.3 | 23±3 | 24±4 | Cardiopulmonary 36; Diabetes 3; Stomach ulcer 3; Previous surgeries 50 | Cardiopulmonary 39; Diabetes 1; Stomach ulcer 3; Previous surgeries 50 | Ileal pouch-anal 11; Low colorectal 66; Coloanal 18 | Ileal pouch-anal 8; Low colorectal 66; Coloanal 17 | Rectal cancer 61; FAP 3; IBD 6; Endometriosis 20; Other 5 | Rectal cancer 60; FAP 2; IBD 5; Endometriosis 20; Other 4 | RT 37 (38.9)  ChT 18 (18.9) | RT 37 (40.6) ChT 14 (15.4) |
| **Lasithiotakis K. 2016** | 16 | 10 | 10:6 | 5:5 | 63 (IQR 24) | 61 (IQR 24) | NR | NR | NR | NR | Colorectal 12; Ileal pouch anal 4 | Colorectal 9; Ileal pouch anal 1 | Rectal cancer 7; Benign disease 9 | Rectal cancer 5; Benign disease 5 | ChRT 4 (25) | ChRT 2 (20) |
| **Danielsen A.K. 2017**  **(Park J. 2018)¹**  **(Park J. 2020)²**  **(Keane C. 2019)¹** | 55 | 57 | 24:31 | 36:21 | 67±11.5 | 67±10.5 | 24±3.7 | 23±4 | 23 (42)  Ischemic Heart Disease 5  Hypertension 17  COPD 2  Renal Disease 0  Other 9 | 24 (42)  Ischemic Heart Disease 8  Hypertension 13  COPD 2  Renal Disease 0  Other 4 | NR | NR | Rectal cancer 55 | Rectal cancer 57 | RT 16 (29) | RT 16 (29) |
| **Kłęk S. 2018** | 29 | 29 | 11:18 | 13:16 | 55.7±12.2 | 56.2±12.5 | NR | NR | NR | NR | Colorectal 29 | Colorectal 29 | Rectal cancer 29 | Rectal cancer 29 | ChRT 29 (100) | ChRT 29 (100) |
| **Gallyamov E.A. 2019** | 31 | 34 | 17:14 | 13:21 | 62±11.5 | 67±10.5 | 24±4 | 23±4.2 | NR | NR | NR | NR | Rectal cancer 31 | Rectal cancer 34 | NR | NR |
| **Bausys A. 2019**  **(Dulskas A. 2021)³** | 43 | 38 | 25:18 | 18:25 | 65±4 | 66±2.5 | NR | NR | 31 (72.1)  Cardiac disease 21  Diabetes 6  Pulmonary disease 1 | 27 (71.1)  Cardiac disease 22  Diabetes 3  Pulmonary disease 1 | NR | NR | Rectal cancer 43 | Rectal cancer 38 | ChRT 20 (46.5) | ChRT 19 (50) |
| **Elsner A. 2021** | 37 | 34 | 21:16 | 26:8 | 67±11.7 | 67±9.7 | 25.4±3.7 | 25.2±4.8 | Diabetes 5 | Diabetes 4 | Colorectal/Coloanal 37 | Colorectal/Coloanal 34 | Rectal cancer 37 | Rectal cancer 34 | ChRT 13 (35) | ChRT 12 (35) |
| **Total** | **306** | **293** | **152:154**  **(49.6:50.4)** | **153:145**  **(52.2:47.8)** | **62.5±4.3** | **62.8±5.1** | **24.1±0.9** | **23.8±1.0** | **Cardiopulmonary 82 (35.6)**  **Diabetes 14 (6.1)**  **Other 12 (1.7)**  **Previous Surg 50 (21.7)** | **Cardiopulmonary 85 (38.6)**  **Diabetes 8 (3.6)**  **Other 7 (3.2)**  **Previous Surg 50 (22.7)** | **Colorectal/anal 162 (91.5)**  **Ileal pouch anal 15 (8.5)** | **Colorectal/anal 155 (94.5)**  **Ileal pouch anal 9 (5.5)** | **Rectal cancer 263 (85.9)** | **Rectal cancer 257 (87.7)** | **Rt 53 (19.2)**  **ChT 18 (6.5)**  **ChRT 66 (24)** | **Rt 53 (20.8)**  **ChT 14**  **(5.4)**  **ChRT 62**  **(23.9)** |

¹ Park J. 2018 and Keane C. 2019 are post-hoc analyses of the EASY trial (Danielson AK. 2017) focused on health-related quality of life and functional outcomes

² Park J. 2020 is a post-hoc analysis of the EASY trial (Danielsen AK. 2017) focused on costs

³ Duskas A. 2021 is a post-hoc analysis of the RCT by Bausys A. 2019 focused on quality of life outcomes and bowel function

RCT= Randomized Controlled Trial; ASA= American Association of Anesthesiologists; BMI= Body Mass Index; TME= Total Mesorectal Excision; IQR= Interquartile Range; NR= Not Reported; FAP= Familial Adenomatous Polyposis; IBD= Inflammatory Bowel Disease; ChT= Chemotherapy; RT= Radiotherapy; ChRT= Chemoradiotherapy; COPD= Chronic Obstructive Pulmonary Disease
