# Supplementary material for: Early versus delayed defunctioning ileostomy closure after low anterior resection for rectal cancer: a meta-analysis and trial sequential analysis of safety and functional outcomes
Source: Int J Colorectal Dis. 2022 Feb 21;37(4):737–56. doi: 10.1007/s00384-022-04106-w (PMC8860143; doi:10.1007/s00384-022-04106-w)
Supplement: Supplementary file 3 — Supplementary file3 (Suppl. Digit. Content. Table 2. Study characteristics - Evidence assessment DOC 332 KB) [file 384_2022_4106_MOESM3_ESM.doc]

**Supplementary Table_2. Study characteristics**

**Author:** Alves A. **Year:** 2008  **Journal:** British Journal of Surgery

| **Setting** Patients with benign or malignant disease requiring elective rectal resection with low anastomosis and diverting ileostomy. |  |  |
| --- | --- | --- |
| **Funding sources** Assistance Publique-Hopitaux de Paris (PHRC AOM 00154). |  |  |
| **Study Design** Randomized controlled trial. |  |  |
| **Participants** 186 patients (95 in the early closure group and 91 in the late closure group). | **Country** France |  |
|  | **Number randomly assigned** Randomization in blocks of 8, stratified by center. 190 patients were randomized. |  |
|  | **Post-randomization dropouts** 4 patients withdrew their consent immediately after randomization. |  |
|  | **Revised sample size** 186 patients. |  |
|  | **Mean age** 58y in the early closure group; 56y in the late closure group. |  |
|  | **Female patients** 51 patients in the early closure group; 49 patients in the late closure group. |  |
|  | **Inclusion criteria** All patients over 18 years old with benign or malignant disease requiring elective rectal resection with a low anastomosis (7 cm or less above the anal verge) and diverting ileostomy. |  |
| **Interventions** If there was no radiological sign of anastomotic leakage after 7 days from the rectal resection, patients were randomized to early closure (on day 8), or late closure (on day 60). |  |  |
| **Outcomes** Primary endpoints were morbidity and mortality rates within 90 days of proctectomy. The secondary endpoints were total hospital stay for all procedure, functional results and quality of life at 12 months. All patients were followed up at 1, 2, 3, 6 and 12 months after rectal resection. |  |  |
| **Risk of bias** *(Revised Cochrane risk-of-bias tool for randomized trials - RoB 2)* |  |  |
| **Outcome(s) being assessed for the risk of bias:** Postoperative morbidity and mortality 90 days after the initial resection. |  |  |
| **Domain: Randomization process** | **Authors' Response** |  |
| 1.1 Was the allocation sequence random? | Yes |  |
| 1.2 Was the allocation sequence concealed until participants were enrolled and assigned to interventions? | Yes |  |
| 1.3 Did baseline differences between intervention groups suggest a problem with the randomization process? | No |  |
| ***Risk-of-bias judgement*** | **LOW** |  |
| ***Domain: Deviations from the intended interventions (effect of assignment to intervention)*** |  |  |
| 2.1 Were participants aware of their assigned intervention during the trial? | No information |  |
| 2.2 Were carers and people delivering the interventions aware of participants' assigned intervention during the trial? | Partially yes |  |
| 2.3 If Y/PY/NI to 2.1 or 2.2: Were there deviations from the intended intervention that arose because of the trial context? | No |  |
| 2.4 If Y/PY to 2.3: Were these deviations likely to have affected the outcome? |  |  |
| 2.5 If Y/PY/NI to 2.4: Were these deviations from intended intervention balanced between groups? |  |  |
| 2.6 Was an appropriate analysis used to estimate the effect of assignment to intervention? | Yes |  |
| 2.7 If N/PN/NI to 2.6: Was there potential for a substantial impact (on the result) of the failure to analyse participants in the group to which they were randomized? |  |  |
| **Risk-of-bias judgement** | **SOME CONCERNS** |  |
| ***Domain: Deviations from the intended interventions (effect of adhering to intervention)*** |  |  |
| 3.1 Were participants aware of their assigned intervention during the trial? | No information |  |
| 3.2 Were carers and people delivering the interventions aware of participants' assigned intervention during the trial? | Partially yes |  |
| 3.3 If Y/PY/NI to 3.1 or 3.2: Were important non-protocol interventions balanced across intervention groups? | No information |  |
| 3.4 Were there failures in implementing the intervention that could have affected the outcome? | No |  |
| 3.5 Was there non-adherence to the assigned intervention regimen that could have affected participants’ outcomes? | No |  |
| 3. 6 If N/PN/NI to 3.3, or Y/PY/NI to 3.4 or 3.5: Was an appropriate analysis used to estimate the effect of adhering to the intervention? | Yes |  |
| ***Risk-of-bias judgement*** | **LOW** |  |
| ***Domain: Missing outcome data*** |  |  |
| 4.1 Were data for this outcome available for all, or nearly all, participants randomized? | Yes |  |
| 4.2 If N/PN/NI to 4.1: Is there evidence that the result was not biased by missing outcome data? |  |  |
| 4.3 If N/PN to 4.2: Could missingness in the outcome depend on its true value? |  |  |
| 4.4 If Y/PY/NI to 4.3: Is it likely that missingness in the outcome depended on its true value? |  |  |
| ***Risk-of-bias judgement*** | **LOW** |  |
| ***Domain: Measurement of the outcome*** |  |  |
| 5.1 Was the method of measuring the outcome inappropriate? | No |  |
| 5. 2 Could measurement or ascertainment of the outcome have differed between intervention groups? | No |  |
| 5.3 If N/PN/NI to 5.1 and 5.2: Were outcome assessors aware of the intervention received by study participants? | Partially yes |  |
| 5.4 If Y/PY/NI to 5.3: Could assessment of the outcome have been influenced by knowledge of intervention received? | No |  |
| 5.5 If Y/PY/NI to 5.4: Is it likely that assessment of the outcome was influenced by knowledge of intervention received? |  |  |
| ***Risk-of-bias judgement*** | **LOW** |  |
| ***Domain: Selection of the reported results*** |  |  |
| 6.1 Were the data that produced this result analysed in accordance with a pre-specified analysis plan that was finalized before unblinded outcome data were available for analysis? | Partially yes |  |
| 6.2 Is the numerical result being assessed likely to have been selected, on the basis of the results, from multiple eligible outcome measurements (e.g. scales, definitions, time points) within the outcome domain? | No |  |
| 6.3 Is the numerical result being assessed likely to have been selected, on the basis of the results, from multiple eligible analyses of the data? | No |  |
| ***Risk-of-bias judgement*** | **LOW** |  |
| ***Overall Risk-of-bias*** | **LOW RISK-OF-BIAS** |  |
| **Study results Morbidity:** 31% in the early group vs. 38% in the late group (P= 0.254)  **Mortality:** No deaths within 90 days  **Overall surgical complications:** 15% in the early group vs. 15% in the late group (P= 1.000)  **Reoperation:** 8% in the early group vs. 8% in the late group (P= 1.000)  **Wound complications:** 19% in the early group vs. 5% in the late group (P= 0.007)  **Small bowel obstruction:** 3% in the early group vs. 16% in the late group (P= 0.002)  **Medical complications:** 5% in the early group vs. 15% in the late group (P= 0.021)  **Hospital stay (days):** 16 (6-59) in the early group vs. 18 (9-262) in the late group (P= 0.013)  **Functional outcomes:** Similar in both groups |  |  |

**Author:** Lasithiotakis K. **Year:** 2016  **Journal:** World Journal of Surgery

| **Setting Patients** (under a single consultant) having a defunctioning ileoastomy during a low rectal or anal anastomosis for benign disease/cancer. |  |  |
| --- | --- | --- |
| **Funding sources** Not reported. |  |  |
| **Study design** Randomized Controlled Trial. |  |  |
| **Participants** 26 patients randomly assigned to undergo early closure (n= 16) or conventional closure (n= 10). | **Country** United Kingdom. |  |
|  | **Number randomly assigned** 26 patients. |  |
|  | **Post-randomization dropouts** None. |  |
|  | **Revised sample size** 26 patients. |  |
|  | **Mean age** 63y in the early closure group; 61y in the late closure group. |  |
|  | **Female patients** 6 patients in the early closure group; 5 patients in the late closure group. |  |
|  | **Inclusion criteria** All consecutive patients (under a single colorectal consultant) having a defunctioning ileostomy during a low rectal or anal anastomosis and with satisfactory gastrografin enema on postoperative day six. |  |
| **Interventions** The early reversal group had the ileostomy reversed on day 7 following the rectal resection; the late reversal group had the ileostomy reversed after an interval of 8 weeks. |  |  |
| **Outcomes** Postoperative complications, Duration of operation (min), ease of abdominal wall closure (VAS), Ease of closure (VAS), Total length of hospital stay (days), Cost of stoma care. |  |  |
| **Risk of bias** *(Revised Cochrane risk-of-bias tool for randomized trials - RoB 2)* |  |  |
| **Outcome(s) being assessed for the risk of bias:** Postoperative complications and duration of the operation. |  |  |
| **Domain: Randomization process** | **Authors' Response** |  |
| 1.1 Was the allocation sequence random? | Yes |  |
| 1.2 Was the allocation sequence concealed until participants were enrolled and assigned to interventions? | Yes |  |
| 1.3 Did baseline differences between intervention groups suggest a problem with the randomization process? | Partially yes |  |
| ***Risk-of-bias judgement*** | **LOW** |  |
| **Domain: Deviations from the intended interventions (*effect of assignment to intervention*)** |  |  |
| 2.1 Were participants aware of their assigned intervention during the trial? | Yes |  |
| 2.2 Were carers and people delivering the interventions aware of participants' assigned intervention during the trial? | No information |  |
| 2.3 If Y/PY/NI to 2.1 or 2.2: Were there deviations from the intended intervention that arose because of the trial context? | No |  |
| 2.4 If Y/PY to 2.3: Were these deviations likely to have affected the outcome? |  |  |
| 2.5 If Y/PY/NI to 2.4: Were these deviations from intended intervention balanced between groups? |  |  |
| 2.6 Was an appropriate analysis used to estimate the effect of assignment to intervention? | Yes |  |
| 2.7 If N/PN/NI to 2.6: Was there potential for a substantial impact (on the result) of the failure to analyse participants in the group to which they were randomized? |  |  |
| ***Risk-of-bias judgement*** | **LOW** |  |
| ***Domain: Deviations from the intended interventions (effect of adhering to intervention)*** |  |  |
| 3.1 Were participants aware of their assigned intervention during the trial? | Yes |  |
| 3.2 Were carers and people delivering the interventions aware of participants' assigned intervention during the trial? | No information |  |
| 3.4 If Y/PY/NI to 3.1 or 3.2: Were important non-protocol interventions balanced across intervention groups? | Yes |  |
| 3.5 Were there failures in implementing the intervention that could have affected the outcome? | No |  |
| 3.6 Was there non-adherence to the assigned intervention regimen that could have affected participants’ outcomes? | No |  |
| 3.7 If N/PN/NI to 3.3, or Y/PY/NI to 3.4 or 3.5: Was an appropriate analysis used to estimate the effect of adhering to the intervention? | Yes |  |
| ***Risk-of-bias judgement*** | **SOME CONCERNS** |  |
| ***Domain: Missing outcome data*** |  |  |
| 4.1 Were data for this outcome available for all, or nearly all, participants randomized? | Yes |  |
| 4.2 If N/PN/NI to 4.1: Is there evidence that the result was not biased by missing outcome data? |  |  |
| 4.3 If N/PN to 4.2: Could missingness in the outcome depend on its true value? |  |  |
| 4.4 If Y/PY/NI to 4.3: Is it likely that missingness in the outcome depended on its true value? |  |  |
| ***Risk-of-bias judgement*** | **LOW** |  |
| ***Domain: Measurement of the outcome*** |  |  |
| *5.1 Was the method of measuring the outcome inappropriate?* | No |  |
| *5.2 Could measurement or ascertainment of the outcome have differed between intervention groups?* | No |  |
| *5.3 If N/PN/NI to 5.1 and 5.2: Were outcome assessors aware of the intervention received by study participants?* | Yes |  |
| *5.4 If Y/PY/NI to 5.3: Could assessment of the outcome have been influenced by knowledge of intervention received?* | Yes |  |
| *5.5 If Y/PY/NI to 5.4:* *Is it likely that assessment of the outcome was influenced by knowledge of intervention received?* | No |  |
| ***Risk-of-bias judgement*** | **LOW** |  |
| ***Domain: Selection of the reported results*** |  |  |
| 6.1 Were the data that produced this result analysed in accordance with a pre-specified analysis plan that was finalized before unblinded outcome data were available for analysis? | No information |  |
| 6.2 Is the numerical result being assessed likely to have been selected, on the basis of the results, from multiple eligible outcome measurements (e.g. scales, definitions, time points) within the outcome domain? | No |  |
| 6.3 Is the numerical result being assessed likely to have been selected, on the basis of the results, from multiple eligible analyses of the data? | Yes |  |
| ***Risk-of-bias judgement*** | **SOME CONCERNS** |  |
| **Overall Risk-of-bias** | **SOME CONCERNS** |  |
| **Study results Postoperative complications:** 25% in the early group vs. 10% in the late group  **Duration of operation (min):** 20 in the early group vs. 40 in the late group  **Ease of abdominal wall closure (VAS):** 80 in the early group vs. 38 in the late group  **Ease of stoma closure (VAS):** 83 in the early group vs. 35 in the late group  **Total length of hospital stay (days):** 14 in the early group vs. 14.5 in the late group  **Cost of stoma care (£):** 27 in the early group vs. 311 in the late group |  |  |

**Author:** Danielsen AK. **Year:** 2017  **Journal:** Annals of Surgery

| **Setting** Patients undergoing rectal resection for cancer with creation of a temporary ileostomy. |  |  |
| --- | --- | --- |
| **Funding sources** Not reported. |  |  |
| **Study design** Randomized Controlled Trial. |  |  |
| **Participants** 112 patients were included. 55 patients in the early and 57 patients in the late closure group. | **Country** Denmark, Sweden. |  |
|  | **Number randomly assigned** 127 patients were randomized and out of these another 15 patients were excluded. In summary, 112 patients were included and completed the follow-up. |  |
|  | **Post-randomization dropouts** 15 patients. |  |
|  | **Revised sample size** 112 patients. |  |
|  | **Mean age** 67y in the early closure group; 67y in the late closure group. |  |
|  | **Female patients** 31 patients in the early closure group and 21 patients in the late closure group. |  |
|  | **Inclusion criteria** >18 y/o patients without any clinical sign of postoperative complications after the index operation (infections or clinical signs of leakage) were invited to participate and after informed consent went through further investigation with a contrast CT scan or a flexible endoscopy or both. This was performed 6 to 8 days after stoma creation to make sure that no patients with signs of anastomotic leakage were included. |  |
| **Interventions** Patients without any adverse signs after rectal resection were invited to participate, and after informed consent went through further investigation of the anastomosis with a contrast CT scan or a flexible endoscopy of the rectum, or both. This was performed 6 to 8 days after stoma creation. Patients in the intervention group underwent early closure (8-13 days after stoma creation); patients in the control group underwent late closure (>12 weeks after stoma creation). |  |  |
| **Outcomes** The primary endpoint was the mean number of complications after the index operation and up to 12 months. |  |  |
| **Risk of bias** *(Revised Cochrane risk-of-bias tool for randomized trials - RoB 2)* |  |  |
| **Outcome(s) being assessed for the risk of bias:** Postoperative complications up to 12 months follow-up. |  |  |
| **Domain: Randomization process** | **Authors' Response** |  |
| 1.1 Was the allocation sequence random? | Yes |  |
| 1.2 Was the allocation sequence concealed until participants were enrolled and assigned to interventions? | Yes |  |
| 1.3 Did baseline differences between intervention groups suggest a problem with the randomization process? | No |  |
| ***Risk-of-bias judgement*** | **LOW** |  |
| **Domain: Deviations from the intended interventions (*effect of assignment to intervention*)** |  |  |
| 2.1 Were participants aware of their assigned intervention during the trial? | Yes |  |
| 2.2 Were carers and people delivering the interventions aware of participants' assigned intervention during the trial? | Partially yes |  |
| 2.3 If Y/PY/NI to 2.1 or 2.2: Were there deviations from the intended intervention that arose because of the trial context? | No |  |
| 2.4 If Y/PY to 2.3: Were these deviations likely to have affected the outcome? |  |  |
| 2.5 If Y/PY/NI to 2.4: Were these deviations from intended intervention balanced between groups? |  |  |
| 2.6 Was an appropriate analysis used to estimate the effect of assignment to intervention? | Yes |  |
| 2.7 If N/PN/NI to 2.6: Was there potential for a substantial impact (on the result) of the failure to analyse participants in the group to which they were randomized? |  |  |
| ***Risk-of-bias judgement*** | **LOW** |  |
| Domain: Deviations from the intended interventions (effect of adhering to intervention) | Yes |  |
| 3.1 Were participants aware of their assigned intervention during the trial? | Partially yes |  |
| 3.2 Were carers and people delivering the interventions aware of participants' assigned intervention during the trial? | Yes |  |
| 3.3 If Y/PY/NI to 3.1 or 3.2: Were important non-protocol interventions balanced across intervention groups? |  |  |
| 3.4 Were there failures in implementing the intervention that could have affected the outcome? |  |  |
| 3.5 Was there non-adherence to the assigned intervention regimen that could have affected participants’ outcomes? | No |  |
| 3.6 If N/PN/NI to 2.3, or Y/PY/NI to 3.4 or 3.5: Was an appropriate analysis used to estimate the effect of adhering to the intervention? | No |  |
| ***Risk-of-bias judgement*** | **SOME CONCERNS** |  |
| ***Domain: Missing outcome data*** |  |  |
| 4.1 Were data for this outcome available for all, or nearly all, participants randomized? | Yes |  |
| 4.2 If N/PN/NI to 4.1: Is there evidence that the result was not biased by missing outcome data? |  |  |
| 4.3 If N/PN to 4.2: Could missingness in the outcome depend on its true value? |  |  |
| 4.4 If Y/PY/NI to 4.3: Is it likely that missingness in the outcome depended on its true value? |  |  |
| ***Risk-of-bias judgement*** | **LOW** |  |
| ***Domain: Measurement of the outcome*** |  |  |
| 5.1 Was the method of measuring the outcome inappropriate? | No |  |
| 5.2 Could measurement or ascertainment of the outcome have differed between intervention groups? | No |  |
| 5.3 If N/PN/NI to 5.1 and 5.2: Were outcome assessors aware of the intervention received by study participants? | Partially yes |  |
| 5.4 If Y/PY/NI to 5.3: Could assessment of the outcome have been influenced by knowledge of intervention received? | No |  |
| 5.5 If Y/PY/NI to 5.4: Is it likely that assessment of the outcome was influenced by knowledge of intervention received? |  |  |
| ***Risk-of-bias judgement*** | **LOW** |  |
| ***Domain: Selection of the reported results*** |  |  |
| *6.1 Were the data that produced this result analysed in accordance with a pre-specified analysis plan that was finalized before unblinded outcome data were available for analysis?* | Yes |  |
| *6.2 Is the numerical result being assessed likely to have been selected, on the basis of the results, from multiple eligible outcome measurements (e.g. scales, definitions, time points) within the outcome domain?* | No |  |
| *6.3 Is the numerical result being assessed likely to have been selected, on the basis of the results, from multiple eligible analyses of the data?* | No |  |
| ***Risk-of-bias judgement*** | **LOW** |  |
| **Overall Risk-of-bias** | **LOW RISK-OF-BIAS** |  |
| **Study results Complications after index surgery up to 12 months follow-up:** 1.2% in the early group vs. 2.9% in the late group (P< 0.0001). |  |  |

**Author:** Klek S. **Year:** 2018  **Journal:** Videosurgery and Other Miniinvasive Techniques

| **Setting** Adult patients who underwent low anterior resection and defunctioning loop ileostomy for rectal carcinoma. |  |  |
| --- | --- | --- |
| **Funding sources Not reported.** |  |  |
| **Study design:** Randomized Controlled Trial. |  |  |
| **Participants** 58 adult rectal cancer patients with defunctioning loop ileostomy created during the low anterior resection, randomly assigned to two study groups: early closure (n= 29), and late closure (n= 29). | **Country** Poland. |  |
|  | **Number randomly assigned** 58 patients. |  |
|  | **Post-randomization dropouts** None. |  |
|  | **Revised sample size** 58 patients. |  |
|  | **Mean age** 55.7y in the early closure group; 56.2y in the late closure group. |  |
|  | **Female patients** 11 patients in the early closure group and 13 patients in the late closure group. |  |
|  | **Inclusion criteria** Patients ≥18 years of age who had undergone anterior resection of the rectum with protective loop ileostomy for rectal adenocarcinoma. |  |
| **Interventions** Early closure group: the patient was re-admitted to the hospital and the ileostomy was closed 14 days after the discharge after the primary resection; Late closure group: the patient was re-admitted to the hospital and the ileostomy was closed 30 days after the termination of the adjuvant chemotherapy (approximately 7 months after the primary resection). |  |  |
| **Outcomes** Primary endpoints: postoperative complications; Secondary endpoints: time to start adjuvant chemotherapy after the primary operation, time to first flatus, time to first defecation, length of hospital stay, readmission, duration of surgery, perioperative blood loss, number of stoma treatment, duration of living with stoma, cost of stoma treatment period. |  |  |
| **Risk of bias** *(Revised Cochrane risk-of-bias tool for randomized trials - RoB 2)* |  |  |
| **Outcome(s) being assessed for the risk of bias:** Time to start adjuvant chemotherapy and complication rate. |  |  |
| **Domain: Randomization process** | **Authors' Response** |  |
| 1.1 Was the allocation sequence random? | Yes |  |
| 1.2 Was the allocation sequence concealed until participants were enrolled and assigned to interventions? | Partially yes |  |
| 1.3 Did baseline differences between intervention groups suggest a problem with the randomization process? | No |  |
| ***Risk-of-bias judgement*** | **LOW** |  |
| **Domain: Deviations from the intended interventions (*effect of assignment to intervention*)** |  |  |
| 2.1 Were participants aware of their assigned intervention during the trial? | Yes |  |
| 2.2 Were carers and people delivering the interventions aware of participants' assigned intervention during the trial? | Yes |  |
| 2.3 If Y/PY/NI to 2.1 or 2.2: Were there deviations from the intended intervention that arose because of the trial context? | No |  |
| 2.4 If Y/PY to 2.3: Were these deviations likely to have affected the outcome? | No |  |
| 2.5 If Y/PY/NI to 2.4: Were these deviations from intended intervention balanced between groups? |  |  |
| 2.6 Was an appropriate analysis used to estimate the effect of assignment to intervention? | No |  |
| 2.7 If N/PN/NI to 2.6: Was there potential for a substantial impact (on the result) of the failure to analyse participants in the group to which they were randomized? | No |  |
| ***Risk-of-bias judgement*** | **HIGH** |  |
| ***Domain: Deviations from the intended interventions (effect of adhering to intervention)*** | **Yes** |  |
| *3.1 Were participants aware of their assigned intervention during the trial?* | Yes |  |
| *3.2 Were carers and people delivering the interventions aware of participants' assigned intervention during the trial?* | Yes |  |
| *3.4 If Y/PY/NI to 3.1 or 3.2: Were important non-protocol interventions balanced across intervention groups?* | No information |  |
| *3.5 Were there failures in implementing the intervention that could have affected the outcome?* | Partially yes |  |
| *3.6 Was there non-adherence to the assigned intervention regimen that could have affected participants’ outcomes?* | No |  |
| *3.7 If N/PN/NI to 2.3, or Y/PY/NI to 3.4 or 3.5: Was an appropriate analysis used to estimate the effect of adhering to the intervention?* | No |  |
| ***Risk-of-bias judgement*** | **SOME CONCERNS** |  |
| ***Domain: Missing outcome data*** |  |  |
| *4.1 Were data for this outcome available for all, or nearly all, participants randomized?* | Yes |  |
| *4.2 If N/PN/NI to 4.1: Is there evidence that the result was not biased by missing outcome data?* | No |  |
| *4.3 If N/PN to 4.2: Could missingness in the outcome depend on its true value?* | No |  |
| *4.4 If Y/PY/NI to 4.3: Is it likely that missingness in the outcome depended on its true value?* |  |  |
| ***Risk-of-bias judgement*** | **LOW** |  |
| ***Domain: Measurement of the outcome*** |  |  |
| 5.1 Was the method of measuring the outcome inappropriate? | No |  |
| 5.2 Could measurement or ascertainment of the outcome have differed between intervention groups? | No |  |
| 5.3 If N/PN/NI to 4.1 and 4.2: Were outcome assessors aware of the intervention received by study participants? | No information |  |
| 5.4 If Y/PY/NI to 4.3: Could assessment of the outcome have been influenced by knowledge of intervention received? | No |  |
| 5.5 If Y/PY/NI to 4.4: Is it likely that assessment of the outcome was influenced by knowledge of intervention received? |  |  |
| ***Risk-of-bias judgement*** | **SOME CONCERNS** |  |
| ***Domain: Selection of the reported results*** |  |  |
| 6.1 Were the data that produced this result analysed in accordance with a pre-specified analysis plan that was finalized before unblinded outcome data were available for analysis? | No information |  |
| 6.2 Is the numerical result being assessed likely to have been selected, on the basis of the results, from multiple eligible outcome measurements (e.g. scales, definitions, time points) within the outcome domain? | No |  |
| 6.3 Is the numerical result being assessed likely to have been selected, on the basis of the results, from multiple eligible analyses of the data? | No |  |
| ***Risk-of-bias judgement*** | **SOME CONCERNS** |  |
| ***Overall Risk-of-bias*** | **SOME CONCERNS** |  |
|  |  |  |
| **Study results Patients with complications:** 10.3% in the early group vs. 13.8% in the late group (P= 0.6873)  **Time to first flatus (median, days):** 2 in the early group vs. 2 in the late group (P= 0.9431)  **Time to first defecation (median, days):** 3 in the early group vs. 3 in the late group (P= 0.5934)  **Length of hospital stay (median, days):** 5 in the early group vs. 5 in the late group (P= 0.6316)  **Readmission:** - in the early group vs. - in the late group  **Duration of surgery (mean, min):** 83.2 in the early group vs. 87.1 in the late group (P= 0.4967)  **Perioperative blood loss (mean, ml):** 15.2 in the early group vs. 17.3 in the late group (P= 0.7142)  **Number of stoma treatment (mean):** 19.1 in the early group vs. 307.6 in the late group (P< 0.001)  **Duration of living with stoma (mean, days):** 17.3 in the early group vs. 278.6 in the late group (P< 0.001)  **Cost of stoma treatment (mean, US Dollars):** 152.9 in the early group vs. 2413.1 in the late group (P< 0.001)  **Time to start adjuvant chemotherapy (mean, days):** 38.7 in the early group vs. 33.2 in the late group (P< 0.001) |  |  |

**Author:** Gallyamov EA. **Year:** 2019  **Journal:** Khirurgiia (Mosk).

| **Setting** Patients who underwent total or partial mesorectal excision for rectal cancer with formation of a defunctioning ileostomy. |  |  |
| --- | --- | --- |
| **Funding sources** Not reported. |  |  |
| **Study design** Randomized Controlled Trial. |  |  |
| **Participants** 65 patients were randomized: 31 patients in the early closure group and 34 patients in the delayed closure group. | **Country** Russia. |  |
|  | **Number randomly assigned** 65 patients. |  |
|  | **Post-randomization dropouts** None. |  |
|  | **Revised sample size** 65 patients. |  |
|  | **Mean age** 62y in the early closure group; 67y in the delayed closure group. |  |
|  | **Female patients** 14 patients in the early closure group and 21 patients in the delayed closure group. |  |
|  | **Inclusion criteria** patients who underwent total or partial mesorectal excision for rectal cancer with formation of a defunctioning ileostomy. |  |
| **Interventions** | Patients in the early closure group underwent ileostomy closure on days 8-13 after rectal excision (CT-proctography or rectoscopy were performed in 8 days after primary surgery to confirm integrity of the colorectal anastomosis); patients in the delayed closure group underwent ileostomy closure >12 weeks after rectal excision. |  |
| **Outcomes** Postoperative morbidity, duration of reconstructive surgery. |  |  |
| **Risk of bias** *(Revised Cochrane risk-of-bias tool for randomized trials - RoB 2)* |  |  |
| **Outcome(s) being assessed for the risk of bias:** Postoperative morbidity and duration of the ileostomy closure procedure. |  |  |
| **Domain: Randomization process** | **Authors' Response** |  |
| 1.1 Was the allocation sequence random? | Yes |  |
| 1.2 Was the allocation sequence concealed until participants were enrolled and assigned to interventions? | Partially yes |  |
| 1.3 Did baseline differences between intervention groups suggest a problem with the randomization process? | No |  |
| ***Risk-of-bias judgement*** | **LOW** |  |
| **Domain: Deviations from the intended interventions (*effect of assignment to intervention*)** |  |  |
| 2.1 Were participants aware of their assigned intervention during the trial? | Yes |  |
| 2.2 Were carers and people delivering the interventions aware of participants' assigned intervention during the trial? | Yes |  |
| 2.3 If Y/PY/NI to 2.1 or 2.2: Were there deviations from the intended intervention that arose because of the trial context? | No |  |
| 2.4 If Y/PY to 2.3: Were these deviations likely to have affected the outcome? |  |  |
| 2.5 If Y/PY/NI to 2.4: Were these deviations from intended intervention balanced between groups? |  |  |
| 2.6 Was an appropriate analysis used to estimate the effect of assignment to intervention? | No |  |
| 2.7 If N/PN/NI to 2.6: Was there potential for a substantial impact (on the result) of the failure to analyse participants in the group to which they were randomized? | No |  |
| ***Risk-of-bias judgement*** | **SOME CONCERNS** |  |
| **Domain: Deviations from the intended interventions (*effect of adhering to intervention*)** |  |  |
| 3.1 Were participants aware of their assigned intervention during the trial? | Yes |  |
| 3.2 Were carers and people delivering the interventions aware of participants' assigned intervention during the trial? | Yes |  |
| 3.3 If Y/PY/NI to 2.1 or 2.2: Were important non-protocol interventions balanced across intervention groups? | Yes |  |
| 3.4 Were there failures in implementing the intervention that could have affected the outcome? | No |  |
| 3.5 Was there non-adherence to the assigned intervention regimen that could have affected participants’ outcomes? | No |  |
| 3.6 If N/PN/NI to 2.3, or Y/PY/NI to 2.4 or 2.5: Was an appropriate analysis used to estimate the effect of adhering to the intervention? | No |  |
| ***Risk-of-bias judgement*** | **HIGH** |  |
| ***Domain: Missing outcome data*** |  |  |
| 4.1 Were data for this outcome available for all, or nearly all, participants randomized? | Partially yes |  |
| 4.2 If N/PN/NI to 3.1: Is there evidence that the result was not biased by missing outcome data? |  |  |
| 4.3 If N/PN to 3.2: Could missingness in the outcome depend on its true value? |  |  |
| 4.4 If Y/PY/NI to 3.3: Is it likely that missingness in the outcome depended on its true value? |  |  |
| ***Risk-of-bias judgement*** | **SOME CONCERNS** |  |
| ***Domain: Measurement of the outcome*** |  |  |
| 5.1 Was the method of measuring the outcome inappropriate? | No |  |
| 5.2 Could measurement or ascertainment of the outcome have differed between intervention groups? | No |  |
| 5.3 If N/PN/NI to 4.1 and 4.2: Were outcome assessors aware of the intervention received by study participants? | Partially yes |  |
| 5.4 If Y/PY/NI to 4.3: Could assessment of the outcome have been influenced by knowledge of intervention received? | No |  |
| 5.5 If Y/PY/NI to 4.4: Is it likely that assessment of the outcome was influenced by knowledge of intervention received? | No |  |
| **Risk-of-bias judgement** | **LOW** |  |
| ***Domain: Selection of the reported results*** |  |  |
| 6.1 Were the data that produced this result analysed in accordance with a pre-specified analysis plan that was finalized before unblinded outcome data were available for analysis? | No information |  |
| 6.2 Is the numerical result being assessed likely to have been selected, on the basis of the results, from multiple eligible outcome measurements (e.g. scales, definitions, time points) within the outcome domain? | No |  |
| 6.3 Is the numerical result being assessed likely to have been selected, on the basis of the results, from multiple eligible analyses of the data? | No |  |
| ***Risk-of-bias judgement*** | **SOME CONCERNS** |  |
| ***Overall Risk-of-bias*** | **SOME CONCERNS** |  |
|  |  |  |
| **Study results Postoperative morbidity:** 6.45% in the early group vs. 5.88% in the delayed group (P= 0.008)  **Duration of reconstructive surgery (mean, min):** 50 in the early group vs. 71 in the delayed group (P= 0.002) |  |  |

**Author:** Bausys A. **Year:** 2019  **Journal:** Journal of Surgical Oncology

| **Setting** Patients with rectal cancer who underwent elective rectal resection with temporary ileostomy. |  |  |
| --- | --- | --- |
| **Funding sources** Not reported |  |  |
| **Study design** Randomized Controlled Trial |  |  |
| **Participants** 86 patients were included. 43 patients were allocated to early stoma closure and 43 patients were allocated to late stoma closure. The study was stopped prematurely by the decision of the trial steering committee after 86 participants were enrolled, due to safety reasons. | **Country** Lithuania. |  |
|  | **Number randomly assigned** 86 patients. |  |
|  | **Post-randomization dropouts** 5 patients allocated to late stoma closure. |  |
|  | **Revised sample size** 81 patients. |  |
|  | **Mean age** 65y in the early closure group; 66 patients in the late closure group. |  |
|  | **Female patients** 18 patients in the early closure group and 25 patients in the late closure group. |  |
|  | **Inclusion criteria** Patients over 18 years old with rectal cancer were screened and included in the study after the elective rectal resection with a temporary loop ileostomy. Patients were included in the study on the 10^th^ postoperative day if they did not meet any of the exclusion criteria. |  |
| **Interventions** To ensure the ileostomy closure safety, the anastomosis was investigated 1 week before with retrograde contrast proctography or/and endoscopy of the rectum. In the early closure group patients underwent ileostomy closure 30 days after creation; in the late closure group patients underwent ileostomy closure 90 days after creation. |  |  |
| **Outcomes** The primary outcome of the study was the number of postoperative complications after ileostomy closure and 30 days afterwards. The secondary endpoints were hospitalization time and 30-day readmission rate. Other prospectively included information were age, sex, comorbidity, duration of surgery, intraoperative technique, time with an ileostomy, the height of rectal tumor, information about neoadjuvant treatment before primary surgery. |  |  |
| **Risk of bias** *(Revised Cochrane risk-of-bias tool for randomized trials - RoB 2)* |  |  |
| **Outcome(s) being assessed for the risk of bias:** 30-day postoperative morbidity following ileostomy closure. |  |  |
| **Domain: Randomization process** | **Authors' Response** |  |
| 1.1 Was the allocation sequence random? | Yes |  |
| 1.2 Was the allocation sequence concealed until participants were enrolled and assigned to interventions? | Yes |  |
| 1.3 Did baseline differences between intervention groups suggest a problem with the randomization process? | No |  |
| ***Risk-of-bias judgement*** | **LOW** |  |
| **Domain: Deviations from the intended interventions (*effect of assignment to intervention*)** |  |  |
| 2.1 Were participants aware of their assigned intervention during the trial? | Yes |  |
| 2.2 Were carers and people delivering the interventions aware of participants' assigned intervention during the trial? | Yes |  |
| 2.3 If Y/PY/NI to 2.1 or 2.2: Were there deviations from the intended intervention that arose because of the trial context? | No |  |
| 2.4 If Y/PY to 2.3: Were these deviations likely to have affected the outcome? |  |  |
| 2.5 If Y/PY/NI to 2.4: Were these deviations from intended intervention balanced between groups? |  |  |
| 2.6 Was an appropriate analysis used to estimate the effect of assignment to intervention? | Partially yes |  |
| 2.7 If N/PN/NI to 2.6: Was there potential for a substantial impact (on the result) of the failure to analyse participants in the group to which they were randomized? |  |  |
| ***Risk-of-bias judgement*** | **SOME CONCERNS** |  |
| ***Domain: Deviations from the intended interventions (effect of adhering to intervention)*** |  |  |
| 3.1 Were participants aware of their assigned intervention during the trial? | Yes |  |
| 3.2 Were carers and people delivering the interventions aware of participants' assigned intervention during the trial? | Yes |  |
| 3.3 If Y/PY/NI to 2.1 or 3.2: Were important non-protocol interventions balanced across intervention groups? | No |  |
| 3.4 Were there failures in implementing the intervention that could have affected the outcome? | No |  |
| 3.5 Was there non-adherence to the assigned intervention regimen that could have affected participants’ outcomes? | No |  |
| 3.6 If N/PN/NI to 2.3, or Y/PY/NI to 2.4 or 2.5: Was an appropriate analysis used to estimate the effect of adhering to the intervention? | No |  |
| ***Risk-of-bias judgement*** | **SOME CONCERNS** |  |
| ***Domain: Missing outcome data*** |  |  |
| 4.1 Were data for this outcome available for all, or nearly all, participants randomized? | Yes |  |
| 4.2 If N/PN/NI to 4.1: Is there evidence that the result was not biased by missing outcome data? |  |  |
| 4.3 If N/PN to 4.2: Could missingness in the outcome depend on its true value? |  |  |
| 4.4 If Y/PY/NI to 4.3: Is it likely that missingness in the outcome depended on its true value? |  |  |
| ***Risk-of-bias judgement*** | **LOW** |  |
| ***Domain: Measurement of the outcome*** |  |  |
| 5.1 Was the method of measuring the outcome inappropriate? | No |  |
| 5.2 Could measurement or ascertainment of the outcome have differed between intervention groups? | No |  |
| 5.3 If N/PN/NI to 4.1 and 5.2: Were outcome assessors aware of the intervention received by study participants? | Yes |  |
| 5.4 If Y/PY/NI to 5.3: Could assessment of the outcome have been influenced by knowledge of intervention received? | No |  |
| 5.5 If Y/PY/NI to 5.4: Is it likely that assessment of the outcome was influenced by knowledge of intervention received? |  |  |
| ***Risk-of-bias judgement*** | **LOW** |  |
| ***Domain: Selection of the reported results*** |  |  |
| 6.1 Were the data that produced this result analysed in accordance with a pre-specified analysis plan that was finalized before unblinded outcome data were available for analysis? | Yes |  |
| 6.2 Is the numerical result being assessed likely to have been selected, on the basis of the results, from multiple eligible outcome measurements (e.g. scales, definitions, time points) within the outcome domain? | No |  |
| 6.3 Is the numerical result being assessed likely to have been selected, on the basis of the results, from multiple eligible analyses of the data? | Yes |  |
| ***Risk-of-bias judgement*** | **LOW** |  |
| **Overall Risk-of-bias** | **LOW RISK-OF-BIAS** |  |
| **Study results Overall 30-day morbidity:** 27.9% in the early group vs. 7.9% in the late group (P= 0.024)  **Postoperative mortality:** 0% in the early group vs. 0% in the late group (P= 1.000)  **Reoperation rate:** 9.3% in the early group vs. 0% in the late group (P= 0.119)  **30-day readmission rate:** 7.0% in the early group vs. 2.6% in the late group (P= 0.618)  **Postoperative hospital stay (mean, days):** 7 in the early group vs. 6 in the late group (P= 0.009)  **Time to ileostomy closure (mean, days):** 34 in the early group vs. 92 in the late group (P= 0.001)  **Operative time (mean, min):** 50 in the early group vs. 50 in the late group (P= 0.648)  **Laparotomy:** 4.7% in the early group vs. 0% in the late group (P= 0.496)  **Ileal resection:** 60.5% in the early group vs. 42.1% in the late group (P= 0.122)  **Unexpected difficulties during surgery:** 39.5% in the early group vs. 5.2% in the late group (P= 0.001) |  |  |

**Author:** Elsner A. **Year:** 2021  **Journal:** Diseases of the Colon & Rectum

| **Setting** Patients who underwent low anterior resection for rectal cancer with a concomitant diverting ileostomy |  |  |
| --- | --- | --- |
| **Funding sources** There was no external research funding for any of the authors |  |  |
| **Study design** Randomized Controlled Trial |  |  |
| **Participants** 71 patients were enrolled. 37 patients were randomized to early closure; 34 patients were randomized to late closure. The trial was stopped for safety concerns after 71 patients were randomized. | **Country** Switzerland |  |
|  | **Number randomly assigned** 72 patients. |  |
|  | **Post-randomization dropouts** 1 patient. |  |
|  | **Revised sample size** 71 patients. |  |
|  | **Mean age** 67y in the early closure group; 67y in the late closure group. |  |
|  | **Female patients** 16 patients in the early closure group and 8 patients in the late closure group. |  |
|  | **Inclusion criteria** Patients undergoing low anterior resection (LAR) for rectal cancer were eligible for participation. Inclusion criteria were age> 18 years, planned anastomosis at 5 cm or less from the anal verge with consecutive fecal diversion via loop ileostomy, and obtained informed consent. |  |
| **Interventions** Patients in the early closure group underwent ileostomy closure 2 weeks after creation; patients in the late closure group underwent ileostomy closure 12 weeks after creation. Patients were randomized if inclusion criteria were met 5 to 8 days after open low anterior resection. Shortly before ileostomy closure, the anastomosis was investigated by palpation, contrast enema via stoma and in some cases by additional proctoscopy. |  |  |
| **Outcomes** Quality of life at 6 weeks (primary endpoint) and 4 months after proctectomy, postoperative complications, operative time, blood loss, tendency of oozing, parastomal and intra-abdominal adhesions, difference in diameters between the two stoma limbs, postoperative recovery, morbidity/safety. |  |  |
| **Risk of bias** *(Revised Cochrane risk-of-bias tool for randomized trials - RoB 2)* |  |  |
| **Outcome(s) being assessed for the risk of bias:** Postoperative morbidity and quality of life 4 months after low anterior resection. |  |  |
| **Domain: Randomization process** | **Authors' Response** |  |
| 1.1 Was the allocation sequence random? | Yes |  |
| 1.2 Was the allocation sequence concealed until participants were enrolled and assigned to interventions? | Yes |  |
| 1.3 Did baseline differences between intervention groups suggest a problem with the randomization process? | No |  |
| ***Risk-of-bias judgement*** | **LOW** |  |
| ***Domain: Deviations from the intended interventions (effect of assignment to intervention)*** |  |  |
| 2.1 Were participants aware of their assigned intervention during the trial? | Partially yes |  |
| 2.2 Were carers and people delivering the interventions aware of participants' assigned intervention during the trial? | Partially yes |  |
| 2.3 If Y/PY/NI to 2.1 or 2.2: Were there deviations from the intended intervention that arose because of the trial context? | No |  |
| 2.4 If Y/PY to 2.3: Were these deviations likely to have affected the outcome? |  |  |
| 2.5 If Y/PY/NI to 2.4: Were these deviations from intended intervention balanced between groups? |  |  |
| 2.6 Was an appropriate analysis used to estimate the effect of assignment to intervention? |  |  |
| 2.7 If N/PN/NI to 2.6: Was there potential for a substantial impact (on the result) of the failure to analyse participants in the group to which they were randomized? | Yes |  |
| ***Risk-of-bias judgement*** | **SOME CONCERNS** |  |
| ***Domain: Deviations from the intended interventions (effect of adhering to intervention)*** |  |  |
| 3.1 Were participants aware of their assigned intervention during the trial? | **Partially yes** |  |
| 3.2 Were carers and people delivering the interventions aware of participants' assigned intervention during the trial? | Partially yes |  |
| 3.3 If Y/PY/NI to 2.1 or 2.2: Were important non-protocol interventions balanced across intervention groups? | Yes |  |
| 3.4 Were there failures in implementing the intervention that could have affected the outcome? | No |  |
| 3.5 Was there non-adherence to the assigned intervention regimen that could have affected participants’ outcomes? | No |  |
| 3.6 If N/PN/NI to 3.3, or Y/PY/NI to 2.4 or 5.5: Was an appropriate analysis used to estimate the effect of adhering to the intervention? | No |  |
| ***Risk-of-bias judgement*** | **SOME CONCERNS** |  |
| **Domain: Missing outcome data** |  |  |
| 4.1 Were data for this outcome available for all, or nearly all, participants randomized? | Yes |  |
| 4.2 If N/PN/NI to 3.1: Is there evidence that the result was not biased by missing outcome data? |  |  |
| 4.3 If N/PN to 3.2: Could missingness in the outcome depend on its true value? |  |  |
| 4.4 If Y/PY/NI to 3.3: Is it likely that missingness in the outcome depended on its true value? |  |  |
| ***Risk-of-bias judgement*** | **LOW** |  |
| ***Domain: Measurement of the outcome*** |  |  |
| 5.1 Was the method of measuring the outcome inappropriate? | No |  |
| 5.2 Could measurement or ascertainment of the outcome have differed between intervention groups? | No |  |
| 5.3 If N/PN/NI to 4.1 and 5.2: Were outcome assessors aware of the intervention received by study participants? | Partially yes |  |
| 5.4 If Y/PY/NI to 5.3: Could assessment of the outcome have been influenced by knowledge of intervention received? | No |  |
| 5.5 If Y/PY/NI to 4.4: Is it likely that assessment of the outcome was influenced by knowledge of intervention received? |  |  |
| ***Risk-of-bias judgement*** | **LOW** |  |
| ***Domain: Selection of the reported results*** |  |  |
| 6.1 Were the data that produced this result analysed in accordance with a pre-specified analysis plan that was finalized before unblinded outcome data were available for analysis? | Yes |  |
| 6.2 Is the numerical result being assessed likely to have been selected, on the basis of the results, from multiple eligible outcome measurements (e.g. scales, definitions, time points) within the outcome domain? | No |  |
| 6.3 Is the numerical result being assessed likely to have been selected, on the basis of the results, from multiple eligible analyses of the data? | No |  |
| ***Risk-of-bias judgement*** | **LOW** |  |
| **Overall Risk-of-bias** | **LOW RISK-OF-BIAS** |  |
| **Study results Operation time (median, mean):** 130 in the early group vs. 110 in the late group (P= 0.197)  **Blood loss (mean, ml):** 14 in the early group vs. 9 in the late group (P= 0.780)  **Tendency of oozing (VAS):** 28 in the early group vs. 14.5 in the late group (P= 0.011)  **Parastomal adhesions (VAS):** 67 in the early group vs. 47.5 in the late group (P= 0.034)  **Intra-abdominal adhesions (VAS):** 31 in the early group vs. 39 in the late group (P= 0.569)  **Time until first defecation (median, days):** 2 in the early group vs. 2 in the late group (P= 0.190)  **Time until full oral intake (median, days):** 4 in the early group vs. 4 in the late group (P= 0.772)  **Total length of hospital stays (median, days):** 28 in the early group vs. 27 in the late group (P= 0.211)  **Total of minor complications (CD I-II):** 32% in the early group vs. 35 in the late group (P= 1.00)  **Total of major complications (CD III-V):** 16% in the early group vs. 0% in the late group (P= 0.026)  **Re-admission:** 8% in the early group vs. 9% in the late group (P= 1.000)  **Overall morbidity after stoma closure:** 49% in the early group vs. 29% in the late group (P= 0.145)  **Stoma closure failure rate:** 27% in the early group vs. 0% in the late group (P= 0.001)  **Quality of life index at 6 weeks (median, range):** 97 in the early group vs. 108 in the late group (P= 0.139)  **Quality of life index at 4 months (median, range):** 106 in the early group vs. 109 in the late group (P= 0.904) |  |  |
