# Supplementary material for: Early versus delayed defunctioning ileostomy closure after low anterior resection for rectal cancer: a meta-analysis and trial sequential analysis of safety and functional outcomes
Source: Int J Colorectal Dis. 2022 Feb 21;37(4):737–56. doi: 10.1007/s00384-022-04106-w (PMC8860143; doi:10.1007/s00384-022-04106-w)
Supplement: Supplementary file 4 — Supplementary file4 (Suppl. Digit. Content. Table 3. Clinical Outcomes of patients following early and delayed ileostomy closure DOC 36 KB) [file 384_2022_4106_MOESM4_ESM.doc]

**Suppl_Table 3. Clinical Outcomes of patients following early and delayed ileostomy closure**

| **Reference** | **Postoperative Mortality**  **N. (%)** | | **Postoperative Morbidity**  **N. (%)** | | **Leak of Rectal Anastomosis**  **N. (%)** | | **Leak of Ileal Anastomosis**  **N. (%)** | | **Unplanned reoperations**  **N. (%)** | | **Operative Time (stoma closure) Min Mean ± SD or Range** | | **Postoperative Length of Hospital Stay (overall and after stoma closure) Days Mean ± SD or Range** | | **Time to start chemotherapy Mean ± SD** | |
| --- | --- | --- | --- | --- | --- | --- | --- | --- | --- | --- | --- | --- | --- | --- | --- | --- |
|  | **Early** | **Delayed** | **Early** | **Delayed** | **Early** | **Delayed** | **Early** | **Delayed** | **Early** | **Delayed** | **Early** | **Delayed** | **Early** | **Delayed** | **Early** | **Delayed** |
| **Alves A. 2008** | - | - | 29 (30.5) | 35 (38.4) | 6 (6.3) | 8 (8.8) | NR | NR | 8 (8.4) | 7 (7.7) | 94 (32-142)* | 95 (33-142)* | 16 (6-59)* | 18 (9-262)* | NR | NR |
| **Lasithiotakis K. 2016** | - | - | 4 (25) | 1 (10) | - | - | - | - | - | - | 20 (IQR 13) | 40 (IQR 9) | 14 (IQR 3) | 14.5 (IQR 4) | NR | NR |
| **Danielsen A.K. 2017**  **(Park J. 2018)¹**  **(Park J. 2020)²**  **(Keane C. 2019)¹** | - | - | Early complications 4 (7)  3 months 18 (33)  6 months 4 (7)  12 months 8 (15) | Early complications 4 (7)  3 months 26 (46)  6 months 18 (32)  12 months 19 (33) | - | 1 (1.7) | - | - | 5 (9) | 4 (7) | 50 (17-180)* | 71 (31-401)* | 14 (11-42)*  After stoma closure  4 (2-27)* | 14 (7-44)*  After stoma closure  4 (2-28)* | NR | NR |
| **Kłęk S. 2018** | - | - | 3 (10.3) | 4 (13.8) | - | - | - | - | 1 (3.4) | 1 (3.4) | 83.2±15.9^ | 87.1±21.7^ | After stoma closure  5 (4-6)* | After stoma closure  5 (4-5)* | 38.7±5.7 | 33.2±5.8 |
| **Gallyamov E.A. 2019** | - | - | 2 (6.4) | 2 (5.9) | - | - | - | - | 1 (3.2) | 1 (2.9) | 50 (27-126)* | 71 (31-134)* | After stoma closure  4 (2-21)* | After stoma closure  4 (2-28)* | NR | NR |
| **Bausys A. 2019**  **(Dulskas A. 2021)³** | - | - | 12 (27.9) | 3 (7.9) | 1 (2.3) | - | 2 (4.6) | - | 4 (9.3) | - | NR | NR | After stoma closure  7 (6-9)* | After stoma closure  6 (6-7)* | NR | NR |
| **Elsner A. 2021** | - | - | 18 (49) | 12 (35) | 3 (8) | - | 2 (5) | - | 3 (8.1) | - | 130 (60-240)* | 110 (60-257)* | 28 (17-77)* | 27 (17-87)* | NR | NR |
| **Total** | **-** | **-** | **90 (29.4)** | **87 (29.6)** | **10 (3.2)** | **9 (3.1)** | **4 (1.9)** | **-** | **22 (7.1)** | **13 (4.4)** | **71.2±39** | **79.0±24.2** | **18±6.7**  **(overall)**  **5±1.4**  **(stoma closure)** | **18.3±6.0**  **(overall)**  **4.7±0.9**  **(stoma closure)** | **38.7±5.7** | **33.2±5.8** |

¹ Park J. 2018 and Keane C. 2019 are post-hoc analyses of the EASY trial (Danielson AK. 2017) focused on health-related quality of life and functional outcomes

² Park J. 2020 is a post-hoc analysis of the EASY trial (Danielsen AK. 2017) focused on costs

³ Duskas A. 2021 is a post-hoc analysis of the RCT by Bausys A. 2019 focused on quality of life outcomes and bowel function

ª Overall postoperative morbidity at 3-months follow-up

* Median (range)

^ Mean ± Standard Deviation

NR= Not Reported; SD= Standard Deviation; IQR= Interquartile Range
