# Supplementary material for: Early versus delayed defunctioning ileostomy closure after low anterior resection for rectal cancer: a meta-analysis and trial sequential analysis of safety and functional outcomes
Source: Int J Colorectal Dis. 2022 Feb 21;37(4):737–56. doi: 10.1007/s00384-022-04106-w (PMC8860143; doi:10.1007/s00384-022-04106-w)
Supplement: Supplementary file 5 — Supplementary file5 (Suppl. Digit. Content. Table 4. Morbidity Outcomes of patients following early and delayed ileostomy closure DOC 34 KB) [file 384_2022_4106_MOESM5_ESM.doc]

**Supplementary Table 4. Morbidity Outcomes of patients following early and delayed ileostomy closure**

| **Reference** | **Clavien Dindo ≥3 Complications N. (%)** | | **Postoperative Ileus/Small Bowel Obstruction N. (%)** | | **Wound Complications N. (%)** | | **Postoperative Intra-abdominal abscess N. (%)** | | **Postoperative Enterocutaneous fistula N. (%)** | | **Bleeding N. (%)** | | **Stoma-Related Complications**  **N. (%)** | | **Anastomotic Stenosis N. (%)** | | **Other Medical Complications N. (%)** | | **Hospital Readmission N. (%)** | |
| --- | --- | --- | --- | --- | --- | --- | --- | --- | --- | --- | --- | --- | --- | --- | --- | --- | --- | --- | --- | --- |
|  | **Early** | **Delayed** | **Early** | **Delayed** | **Early** | **Delayed** | **Early** | **Delayed** | **Early** | **Delayed** | **Early** | **Delayed** | **Early** | **Delayed** | **Early** | **Delayed** | **Early** | **Delayed** | **Early** | **Delayed** |
| **Alves A. 2008** | NR | NR | 3 (3.1) | 15 (16.4) | 18 (18.9) | 5 (5.5) | - | 2 (2.2) | 5 (5) | 1 (1) | 1 (1.1) | 1 (1.1) | 1 (1.1) | 11 (12.1) | 2 (2.1) | - | 4 (4.2) | 3 (3.3) | NR | NR |
| **Lasithiotakis K. 2016** | NR | NR | 1 (6.2) | 1 (10) | 2 (12.5) | - | - | - | NR | NR | - | - | - | - | - | - | 1 (6.2) | - | NR | NR |
| **Danielsen A.K. 2017**  **(Park J. 2018)¹**  **(Park J. 2020)²**  **(Keane C. 2019)¹** | 14 (25.4)  Early complications 2  3 months 10  6 months 0  12 months 2 | 26 (45.6)  Early complications 4  3 months 11  6 months 3  12 months 8 | 1 (1.8) | 1 (1.7) | 2 (3.6) | - | 3 (5.4) | 2 (3.5) | NR | NR | - | - | 13 (24) | 44 (77) | - | 1 (1.7) | 4 (7.2) | 4 (7.0) | NR | NR |
| **Kłęk S. 2018** | 1 (3.4) | 1 (3.4) | 1 (3.4) | - | 2 (6.9) | 3 (10.3) | - | - | NR | NR | - | 1 (3.4) | - | - | - | - | - | - | NR | NR |
| **Gallyamov E.A. 2019** | 1 (3.2) | 1 (2.9) | - | 1 (2.9) | NR | NR | NR | NR | NR | NR | NR | NR | NR | NR | NR | NR | NR | NR | NR | NR |
| **Bausys A. 2019**  **(Dulskas A. 2021)³** | 5 (11.6) | - | 2 (4.7) | 2 (5.3) | 3 (7.0) | - | 1 (2.3) | - | 1 (2.3) | - | - | - | NR | NR | - | - | 2 (4.7) | 1 (2.6) | 3 (7) | 1 (2.6) |
| **Elsner A. 2021** | 6 (16) | - | 4 (11) | 5 (14) | 3 (8.1) | 2 (6) | 2 (5.4) | - | - | - | - | - | - | 2 (6) | - | - | 6 (16.2) | 3 (9) | 3 (8) | 3 (9) |
| **Total** | **27 (13.8)** | **28 (14.5)** | **12 (3.9)** | **25 (8.5)** | **30 (10.9)** | **10 (3.8)** | **6 (2.2)** | **4 (1.5)** | **6 (3.4)** | **1 (0.6)** | **1 (0.3)** | **2 (0.7)** | **14 (6)** | **57 (25.7)** | **2 (0.7)** | **1 (0.3)** | **17 (6.1)** | **11 (4.2)** | **6 (7.5)** | **4 (5.5)** |

¹ Park J. 2018 and Keane C. 2019 are post-hoc analyses of the EASY trial (Danielson AK. 2017) focused on health-related quality of life and functional outcomes

² Park J. 2020 is a post-hoc analysis of the EASY trial (Danielsen AK. 2017) focused on costs

³ Duskas A. 2021 is a post-hoc analysis of the RCT by Bausys A. 2019 focused on quality of life outcomes and bowel function

NR= Not Reported
