# Supplementary material for: Early versus delayed defunctioning ileostomy closure after low anterior resection for rectal cancer: a meta-analysis and trial sequential analysis of safety and functional outcomes
Source: Int J Colorectal Dis. 2022 Feb 21;37(4):737–56. doi: 10.1007/s00384-022-04106-w (PMC8860143; doi:10.1007/s00384-022-04106-w)
Supplement: Supplementary file 6 — Supplementary file6 (Suppl. Digit. Content. Table 5. Quality of Life, Functional Outcomes and Costs DOC 27 KB) [file 384_2022_4106_MOESM6_ESM.doc]

**Supplementary Table 5. Quality of Life, Functional Outcomes and Costs**

| **Reference** | **Follow-up** | **Quality of Life Mean ± SD** | | **LARS N. (%) or Mean ± SD** | | **Memorial Sloan Kettering Cancer Centre Bowel Function Instrument (MSKCC-BFI)**  **Median (Range)** | | **Costs** | |
| --- | --- | --- | --- | --- | --- | --- | --- | --- | --- |
|  |  | **Early** | **Delayed** | **Early** | **Delayed** | **Early** | **Delayed** | **Early** | **Delayed** |
| **Alves A. 2008*** | 12 months | 111±16.3 | 108±17 | NR | NR | NR | NR | NR | NR |
| **Lasithiotakis K. 2016** | NR | NR | NR | NR | NR | NR | NR | 27 (9 IQR) ^b^ | 311 (108 IQR) ^b^ |
| **Danielsen A.K. 2017**  **(Park J. 2018)¹ ª**  **(Keane C. 2019)¹** | 3 months  6 months  12 months  49 months (Keane) | 75±8.3  66.7±8.3  83.3±10.4 | 66.7±6.2  66.7±4.1  83.3±6.2 | Minor LARS 7 (16.6)  Major LARS 25 (59.5)  Score 31±3.5 | Minor LARS 5 (12.5)  Major LARS 29 (72.5)  Score 34±2.7 | 71±4 | 63±2.5 | 31243 ^c^ | 35303 ^c^ |
| **Kłęk S. 2018** | NR | NR | NR | NR | NR | NR | NR | 152.9±16.3^d^ | 2413.1±759^d^ |
| **Gallyamov E.A. 2019** | NR | NR | NR | NR | NR | NR | NR | NR | NR |
| **Bausys A. 2019**  **(Dulskas A. 2021)ª** | 36 months | 37.2±24.9 | 34.3±16.2 | Minor LARS 6 (23%)  Major LARS 6 (23%) | Minor LARS 6 (24%)  Major LARS 8 (32%) | NR | NR | NR | NR |
| **Elsner A. 2021*** | 6 weeks  4 months | 97±18.7  106±17 | 108±18.2  109±14.7 | NR | NR | NR | NR | NR | NR |

*Gastrointestinal Quality of Life Index (GQLI)

ªEORTC QLQ-C30 Quality of Life

^b^ Stoma care (£ Pound Sterling); ^c^Total costs (Index surgery included, United States Dollars USD); ^d^Stoma care United States Dollars (USD)

¹ Park J. 2018 and Keane C. 2019 are post-hoc analyses of the EASY trial (Danielsen AK. 2017) focused on health-related quality of life and functional outcomes

LARS= Low Anterior Resection Syndrome; IQR= Interquartile Range; SD= Standard Deviation; NR= Not Reported; SE= Standard Error; CI= Confidence Interval
