# Supplementary material for: Early versus delayed defunctioning ileostomy closure after low anterior resection for rectal cancer: a meta-analysis and trial sequential analysis of safety and functional outcomes
Source: Int J Colorectal Dis. 2022 Feb 21;37(4):737–56. doi: 10.1007/s00384-022-04106-w (PMC8860143; doi:10.1007/s00384-022-04106-w)
Supplement: Supplementary file 7 — Supplementary file7 (Digit. Content. Table 6. Results of the Trial Sequential Analysis DOC 22 KB) [file 384_2022_4106_MOESM7_ESM.doc]

**Suppl. Table 6. Results of the Trial Sequential Analysis**

| **Outcome** | **Type of outcome** | **Scenario** | **Risk relative reduction** | **RR** | **α spending adjusted CI** | **Variance** | **Alpha** | **Beta** | **Require IS** | **Actual IS** | **Exceed IS** | **Monitoring boundaries** | **Foutility boundaries** |
| --- | --- | --- | --- | --- | --- | --- | --- | --- | --- | --- | --- | --- | --- |
| Post operative Morbidity | Dichotomous | Scenario 1 | 10% | 0.99 | Ignored due too little information use (3.67%) | 55.85 | 5% | 20% | 16330 | 599 | No | Not crossed | Not crossed |
| Post operative Morbidity | Dichotomous | Scenario 2 | 25% | 0.99 | 0.61 - 1.63 | 55.85 | 5% | 20% | 2478 | 599 | No | Not crossed | Not crossed |
| Leak of rectal anastomosis | Dichotomous | Scenario 1 | 1% | 1.04 | Ignored due too little information use (0%) | 50 | 5% | 20% | 10102024 | 599 | No | Not crossed | Not crossed |
| Leak of rectal anastomosis | Dichotomous | Scenario 2 | 25% | 1.04 | Ignored due too little information use (3.15%) | 50 | 5% | 20% | 28534 | 599 | No | Not crossed | Not crossed |
| Unplanned reoperations | Dichotomous | Scenario 1 | 5% | 1.6 | Ignored due too little information use (0.19%) | 50 | 5% | 20% | 294169 | 573 | No | Not crossed | Not crossed |
| Unplanned reoperations | Dichotomous | Scenario 2 | 25% | 1.6 | 0.11 - 22.54 | 50 | 5% | 20% | 10604 | 573 | No | Not crossed | Not crossed |
